# Thin‐Film Transistor Based Active Taxel for Multimode Tactile Perception and Fused Processing

**DOI:** 10.1002/advs.76190

**Published:** 2026-06-17

**Authors:** Sihao Wu, Zheng Zhou, Aoran Xu, Lutong Wang, Tingchen Yi, Chuanlin Sun, Haotong Zhu, Jiaqi Li, Junchen Dong, Li Zhou, Lifeng Liu, Yimao Cai, Dedong Han, Xing Zhang

**Affiliations:** ^1^ School of Integrated Circuits Peking University Beijing China; ^2^ School of Information & Communication Engineering Beijing Information Science and Technology University Beijing China; ^3^ BOE Technology Group Co., Ltd. Beijing China; ^4^ Beijing Advanced Innovation Center for Integrated Circuits Beijing China

**Keywords:** artificial skin, fingerprint recognition, in‐sensor computing, multimode fused perception, object classification, taxels, thin‐film transistors

## Abstract

Skin is the largest‐area organ for humans and embodied intelligent robots, serving as a critical interface for environmental interaction. Thanks to the well‐organized taxel, dynamic‐taxel‐density and fully body wrapping, human skin works as an efficient tactile system for embodied intelligent robots to emulate. Thin‐film transistor (TFT) technology is a well‐known mature semiconductor process for mass production with the largest‐area substrate, inherently suitable for artificial skin developing. In this work, we propose an active multimode fused (AMF) artificial skin developed from a standard TFT process for intelligent robots. A novel 2T‐1C taxel integrating optical and electrostatic capacitive receptors is developed for cross‐modal feature extraction. A 10×10 AMF artificial skin sample within 1 cm^2^ is fabricated and experimentally validated through Braille perception. The AMF skin has high tactile robustness, retaining 81.7% accuracy in complex fingerprint tasks even with 45% information loss. Moreover, a fully skin‐wrapped dexterous hand integrated with a dynamic‐taxel‐density tactile system is presented, enabling accurate texture‐ and shape‐dependent object recognition with 80% less data movement and 76.6% lower computational cost. The TFT‐based artificial skin paves an approach for the development of embodied intelligent robots with full‐body skin wrapping.

## Graphical abstract

Skin serves as the largest‐area organ for human and embodied intelligent robots, providing tactile interaction. An active multimode fused (AMF) artificial skin is developed using standard TFT processes, featuring 2T‐1C taxels with optical and electrostatic capacitive receptors for cross‐modal sensing. This artificial skin paves an approach for the development of embodied intelligent robots with full‐body skin wrapping.

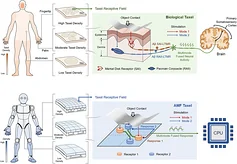

## 1 Introduction

**FIGURE 1:**
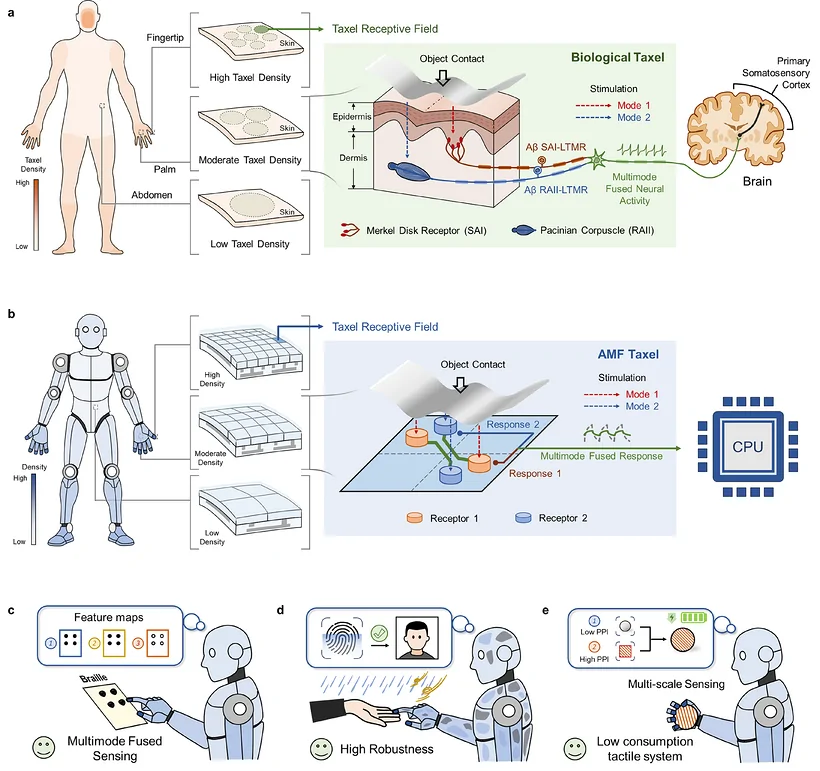
Bioinspired TFT‐based AMF artificial skin for embodied intelligent robots. (a) Schematic of the human tactile system. Biological taxels are non‐uniformly distributed across the body and consist of one or several adjacent receptors. The neural impulses generated by these receptors are pre‐integrated before reaching the central brain. (b) Schematic of the TFT‐based AMF artificial skin. The artificial skins with various resolution are applied to different regions of an embodied intelligent robot. AMF taxels serve as the fundamental units of artificial skin, enabling multimode sensing and early‐stage processing. (c) Schematic of multimode fused sensing enabled by the AMF artificial skin. (d) Schematic of highly robust sensing based on AMF artificial skin under harsh environments. (e) Schematic of the AMF artificial skin‐based dynamic‐taxel‐density tactile system enabling low‐cost object perception.

As the largest organ in the human body, skin serves as a vital interface between the human and the physical world, endowing human with the powerful tactile perception capability [1]. Anatomical studies reveal that the skin contains functional taxels formed by overlapping neighboring receptors of various types, enabling multimode sensing that ensures high tactile robustness against environmental disturbances, even underwater or in harsh weather [1, 2, 3, 4, 5]. Moreover, the density of the taxels varies markedly across body regions, decreasing from approximately 60 taxels per cm^2^ at the fingertips to about 5 taxels per cm^2^ at the abdomen (Figure 1a) [6, 7]. This dynamic‐density supports multi‐scale tactile perception that balances sensitivity with metabolic efficiency. The well‐organized taxel in human skin provides both tactile efficiency and robustness, inspiring the development of artificial skin for intelligent robots.

Serving as the core components of taxels in embodied intelligent robots, tactile receptors have recently been developed in diverse forms, including optical, electrostatic, force, strain, and temperature types. Among them, optical receptors are well‐suited for robot‐object interaction owing to their high spatial resolution and sensing precision. Son et al. proposed a highly sensitive behavioral biometric optical receptor capable of real‐time decoupling touch signals into normal and shear force components from a single high‐resolution image [8]. Wang et al. developed an optical‐based multipoint 3‐axis pressure receptor that enables high‐accuracy sensing of normal and tangential forces, while maintaining a compact form factor and flexibility [9]. Moreover, electrostatic capacitive receptors are widely employed in robot‐human interaction because of their high sensitivity and high‐speed response characteristics. An et al. developed a transparent and flexible mutual‐capacitive tactile receptor array that accurately extracts fingerprint information through electrostatic coupling, offering advantages of high resolution, strong noise resistance, and high‐frequency operation capability [10]. Since a single type of receptor provides limited information about tactile stimuli, multimode perception has become essential for improving robustness and achieving more comprehensive tactile perception [11, 12, 13, 14, 15, 16, 17, 18, 19, 20]. However, the data explosion from numerous receptors strains both computational resources and power efficiency [21], posing big barriers to the commercial viability of fully skin wrapped intelligent robots. In comparison, human skin avoids such a data explosion through a specialized multimode fused perception strategy in taxel [22, 23]. Each taxel preprocesses and integrates tactile signals from its constituent receptors before relaying them to the central brain (Figure 1a). In cognitive science, this strategy enables the human tactile system to capture external object features with minimal data, reducing the workload of brain, especially in dynamic, complex environments [24]. This inspires us to construct an active multimode fused (AMF) taxel for embodied intelligent robots, enabling both robust multimodal perception and early‐stage information processing at the device level.

TFT technology is a well‐developed platform for mass production [25, 26]. Benefiting from advances in the semiconductor display industry, flexible TFT technology has reached Generation‐6 (1500 mm × 1850 mm) and provides numerous off‐the‐shelf fabrication equipment for artificial skin, which is large enough for skin fully‐wrapped intelligence robots. In addition, TFT platform enables the development and integration of diverse receptors that cover multiple physical quantities [27, 28, 29]. Building on this multimodal compatibility, the active characteristics of TFTs further allow in‐sensor signal modulation and preprocessing, making the TFT platform an ideal foundation for constructing AMF taxels. Moreover, TFT possess intrinsic advantages in both device scaling [30] and active circuit design [31, 32], enabling dynamic‐taxel‐density and functional artificial skin design (Figure 1b). Collectively, these attributes make the TFT platform a promising approach for constructing AMF taxels and even large‐area artificial skin in future intelligent robots (Table S1).

In this work, we proposed a bioinspired AMF taxel based on TFT technology for embodied intelligent robots. By leveraging the active properties of TFT and photosensitivity of zinc oxide (ZnO)‐channel, we developed a 2T‐1C AMF taxel integrating optical and electrostatic capacitive receptors. With a specialized spatial arrangement, the AMF taxel realizes cross‐modal Roberts operator for edge extraction. We fabricated a 10×10 AMF artificial skin sample inside 1 cm^2^, and experimentally verified by Braille perception task (Figure 1c). The artificial skin exhibits strong tactile robustness under harsh conditions, maintaining 81.7% fingerprint‐based personal identity verification (PIV) accuracy even under 45% information loss (Figure 1d). Furthermore, a simulated dexterous hand fully covered with dynamic‐taxel‐density artificial skin is presented to evaluate the application potential of such skin in embodied intelligent robots. This system achieves a favorable balance between performance and hardware cost as demonstrated in texture‐shape‐dependent object recognition tasks (Figure 1e).

## 2 Results

### 2.1 Active Multimode Fused Taxel

**FIGURE 2:**
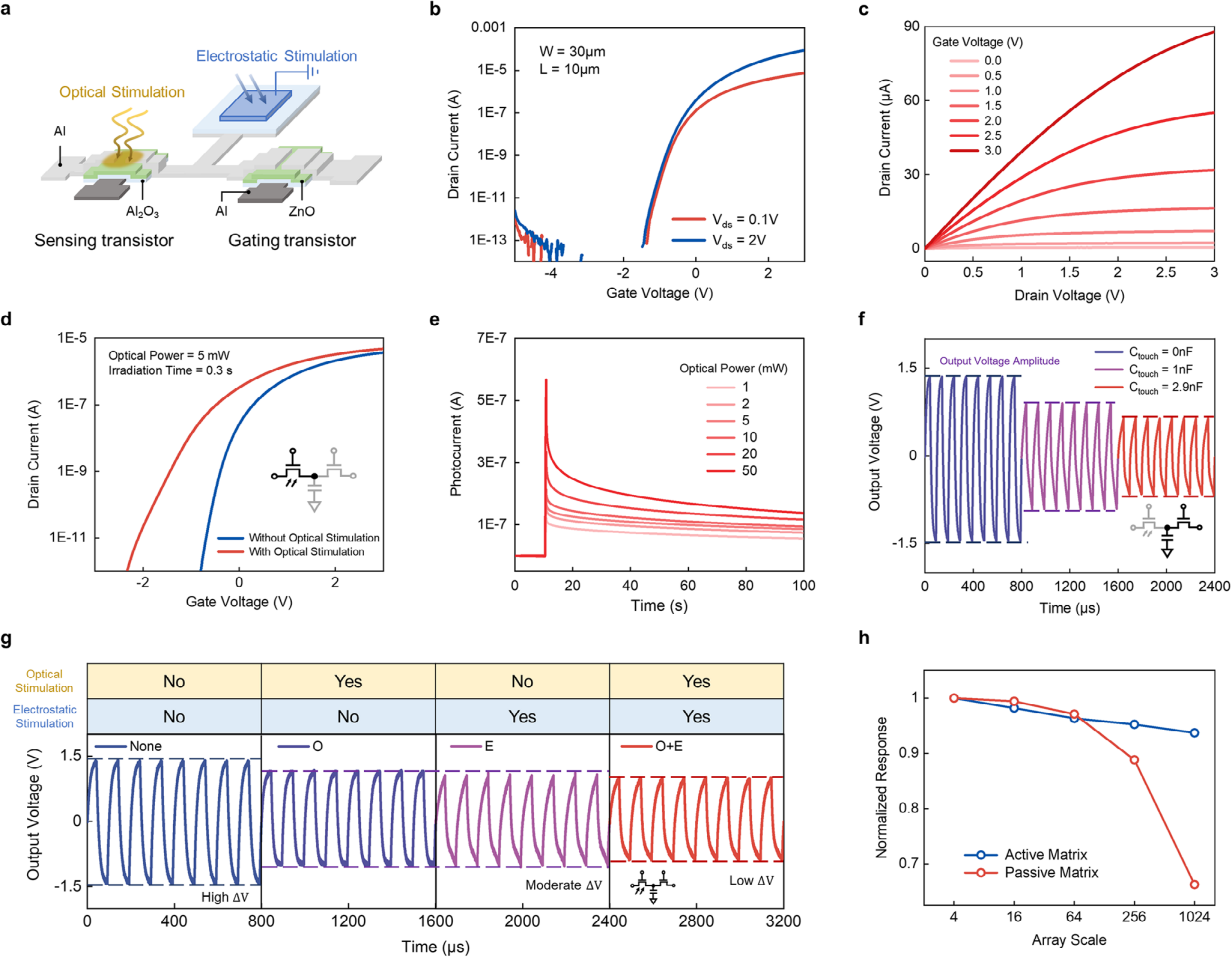
Multimode sensing characteristics of the AMF taxel. (a) Schematic of the AMF taxel, consisting of a 1T optical receptor and a 1T‐1C electrostatic capacitive receptor. (b) Typical transfer characteristic curves of the ZnO TFT. (c) Typical output characteristic curves of the ZnO TFT. (d) Transfer curve of the optical receptor before and after optical stimulation (wavelength of 380 nm, optical power of 5 mW, irradiation time of 0.3 s). The applied V_ds_ is 0.1 V. (e) Photocurrent of the optical receptor under varying optical stimulations (optical power of 1, 2, 5, 10, 20, 50 mW, irradiation time of 0.3 s). The applied V_gs_ and V_ds_ are 0 and 0.1 V, respectively. (f) Output voltage of the electrostatic capacitive receptor under varying electrostatic stimulations (C_touch_ of 0, 1, 2.9 nF, gate voltage of 0 V). (g) Output voltage of the AMF taxel under various stimulations. The optical stimulation is 50 mW for 1 s at 380 nm. The electrostatic stimulation introduces C_touch_ of 1 nF. The applied gate voltage of the sensing transistor and the gating transistor are 0 and 1 V, respectively. (h) Normalized tactile responses of passive tactile array and AMF taxel‐based active tactile array at different scales.

The AMF taxel we proposed consists of a 1T optical receptor and a 1T‐1C electrostatic capacitive receptor (Figure 2a). For mass‐production compatibility, the fabrication process of the unit follows the standard back‐gate TFT technology flow. Aluminum (Al) is used for the gate, source, and drain electrodes; aluminum oxide (Al_2_O_3_) for the gate dielectric, and ZnO for the channel material. Process details are provided in the “Methods” section. Atomic force microscopy and X‐ray photoelectron spectroscopy O 1s spectra reveal that the channel layer exhibits a low root‐mean‐square roughness (Rq) and a high density of oxygen vacancies (Figure S1). Besides, transmission electron microscopy images and energy dispersive spectroscopy mappings (Figure S2) clearly show the well‐formed films of the ZnO TFTs. The typical transfer and output characteristics are shown in Figure 2b,c, respectively. This device exhibits a low off‐state current below 10^−14^ A and a high on/off current ratio exceeding 10^9^, demonstrating high‐performance switching characteristics as an active device.

The ZnO TFT exhibits photosensitive characteristics [33, 34], which is suitable for the implementation of optical receptor. After 380‐nm laser irradiation, the transfer characteristic curve showed a significant negative shift (Figure 2d). This shift is primarily caused by an increase in channel current following irradiation, which is attributed to the directional drift of photogenerated carriers in ZnO under the influence of a lateral electric field. These photogenerated carriers originate from intrinsic excitations in ZnO and the ionization of neutral oxygen vacancies (V_o_) (Note S1 and Figure S3). When a drain‐source voltage of 0.1 V was applied to the ZnO TFTs, the photogenerated carriers were converted to an enhanced photocurrent (Figure 2e). The photocurrent increases with optical power due to the more photogenerated carriers, showing an approximately linear relationship in the range of 1 mW to 50 mW, with a responsivity of approximately 71.44 µA/W. A similar trend is observed with irradiation time, as shown in Figure S4. Furthermore, temperature stability tests demonstrate that the optical receptor maintains consistent performance over the range from 20°C to 80°C, confirming its reliability in various environmental conditions (Figure S5).

Electrostatic is a widely used sensing approach in display panels with passive structure, such as self‐ or mutual‐capacitance screen [10, 35, 36]. Here, we designed an active electrostatic capacitive receptor to enable better signal preprocessing and facilitate larger‐scale integration. The receptor consists of a capacitor for electrostatic sensing and a TFT as the selector. When a conductive object approaches, an additional touch capacitance (C_touch_) is introduced at the source electrode through electrostatic coupling. C_touch_ is proportional to the contact area (Figure S6) and can be precisely detected using a readout circuit (Figure S7). Here, a ±1.5 V, 10 kHz periodic square‐wave was used to scan the receptors and was converted into an output voltage. A larger C_touch_ induced a larger voltage change, attributed to a higher average channel current during the charge–discharge cycle, with a sensitivity of approximately 0.99 V/nF for the electrostatic capacitive receptor. (Figure 2f). This voltage amplitude window broadened with increasing gate voltage, reflecting the current modulation capability of the TFT as an active element (Figure S8). The electrostatic capacitive receptor exhibits a minimum response time of 8.33 µs and strong cycle‐to‐cycle (C2C) stability, demonstrating its potential for practical applications (Figure S9). Additionally, the receptor also shows reliable performance across a temperature range of 20°C–80°C (Figure S10).

The AMF taxel adopts an adder‐like integrated design that enables in‐sensor fusion of multimode stimuli, with both optical and electrostatic stimulations uniformly translated into changes in gating transistor current that jointly influence the output of AMF taxel. Under a ±1.5 V, 10 kHz square‐wave drive, the photocurrent from optical stimulation and the C_touch_ from electrostatic stimulation both enhanced the gating transistor current and consequently reduced the output voltage amplitude (Figure S11). As a result, the unit output decreased significantly in response to either optical stimulation (380 nm, 50 mW, 1 s) or electrostatic stimulation (C_touch_ = 1 nF) and showed an even lower value under dual‐stimulation (Figure 2g). Furthermore, increased optical power or larger C_touch_ leads to additional voltage reductions (Figure S12). Touch‐induced electrostatic coupling with the TFT in the AMF taxel does not significantly affect its output (Figure S13). Since the electrode area of the TFT is much smaller than that of the capacitor, touch‐induced electrostatic coupling on the TFT only causes an overall 0.5% reduction in the output voltage amplitude of the AMF taxel, which is much smaller than the response amplitude to external stimulation and can therefore be neglected. Compared with passive tactile arrays, active tactile arrays based on AMF taxels effectively suppress crosstalk within the array, thereby maintaining sufficiently high responsiveness even at larger array scales (Figure 2h). This makes them more suitable for applications in full‐body artificial skin.

### 2.2 Cross‐Modal Operator and Feature Extraction

**FIGURE 3:**
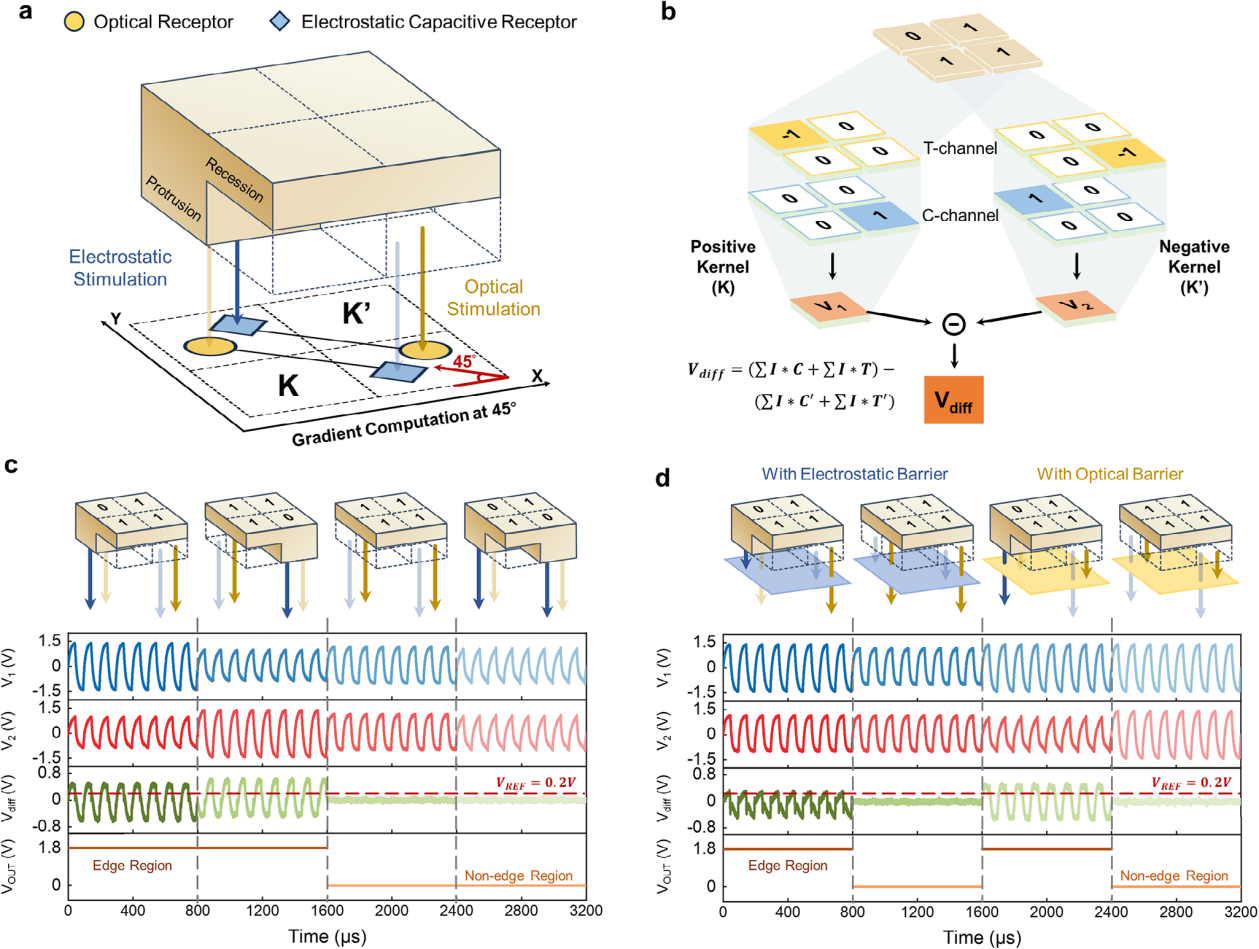
In‐sensor edge extraction with cross‐modal operator (CPO). (a) Schematic of edge extraction in the diagonal (45°) direction using the CPO. Object protrusions induce electrostatic stimulation through direct contact, while recessions allow light transmission, leading to optical stimulation. (b) Configuration of the CPO. The operator consists of a positive and a negative sub‐kernel, comprising a T‐channel and a C‐channel that correspond to optical and electrostatic capacitive receptors in the CPO, respectively. The weights assigned to optical receptors and electrostatic receptors are “−1” and “1”, respectively, while positions without receptors are assigned “0”. Protrusions are encoded as “0” and recessions as “1”. (c) Measure result of the CPO under all possible input patterns in the diagonal direction. Input “0” corresponding to electrostatic stimulation (1 nF). Input “1” corresponding to optical stimulation (380 nm, 50 mW, 1 s). The applied V_SG_ and V_GG_ for each AMF taxel are 0 and 1 V, respectively. (d) The measure result of the CPO under typical input patterns in the diagonal direction with singlemode interferences. These barriers block the corresponding stimulation from reaching the receptors. Other measurement parameters are consistent with c.

Inspired by multimode fused perception strategy in human skin, we designed a hardware‐based cross‐modal operator (CPO). The CPO consists of a pair of AMF taxels arranged in diagonal/anti‐diagonal (45°/135°) directions, enabling gradient computation of the contacted object's morphology (Figure 3a). Mathematically, each AMF taxel represents a 2‐channel kernel, and performs convolution with the contacted object (Figure 3b). The weight of the optical receptor is ‐1, while the electrostatic capacitive receptor is +1, based on their opposite response as the object approaches. A shared‐column differential peripheral circuit is introduced, allowing one AMF taxel to generate completely opposite weights (negative kernel) (Figure S14). The simple differential amplifiers and hysteresis comparators used in the differential peripheral circuit add negligible complexity compared to conventional ADCs. In this way, a CPO for edge extraction is constructed, analogous to the Roberts operator in singlemode scenarios. The CPO's equivalent circuit is shown in Figure S14, and the following equation represents the convolution process:

(1) $$V_{diff}=\left(\sum I*C+\sum I*T\right)\;-\left(\sum I*C^{\prime }+\sum I*T^{\prime }\right)$$

where I is input, C, T belong to the positive kernel, and C’ and T’ belong to the negative kernel. The paired AMF taxels and differential structure offer an additive inverse in cross‐modal scenarios, and more CPOs can be developed through hardware‐algorithm co‐design. Figure 3c demonstrates the measurement result in possible morphological patterns along the 45° direction. Adjacent differing pixels (i.e., one protrusion, one recession) result in a higher V_diff_, with peak amplitudes exceeding 0.5 V; identical pixels (both protrusions / recessions) result in V_diff_ near zero. A comparator with a 0.2 V reference (V_REF_) can reliably separate edges from non‐edges. Furthermore, the CPO can operate even with non‐ideal multi‐value inputs (Figure S15). When the input deviates slightly from the ideal binary case, the V_diff_ amplitude in edge regions decreases, whereas that in non‐edge regions increases. As long as the input fluctuation remains within a tolerable range, a fixed V_REF_ can still distinguish between these two V_diff_ values, thereby enabling reliable edge extraction based on CPO. If the AMF taxel outputs in the CPO are approximated as varying linearly with either C_touch_ or optical power, the tolerable input fluctuation is 1/3. For the actual CPO, the tolerable fluctuation is slightly less than 1/3. Notably, since each pixel undergoes repeated multimode sensing, the operator is inherently immune to singlemode noise. Even when a certain modal stimulus is absent or does not change with the edge, the maximum V_diff_ still exceeds 0.3 V, while non‐edges remain near 0 V (Figure 3d). In such cases, the CPO functions similarly to a standard Roberts operator. Therefore, this cross‐modal CPO can be employed to construct artificial skin capable of robust in‐situ edge extraction. Although using CPO for edge extraction in artificial skin reduces image resolution, it simultaneously serves as a form of information compression. It transforms the original image's rich but redundant data into a smaller amount of high‐information‐density edge information, thereby reducing data movement and processing overhead while still maintaining image recognition accuracy. This trade‐off ultimately enables the realization of a low‐power tactile system.

### 2.3 AMF Artificial Skin and Braille Perception

**FIGURE 4:**
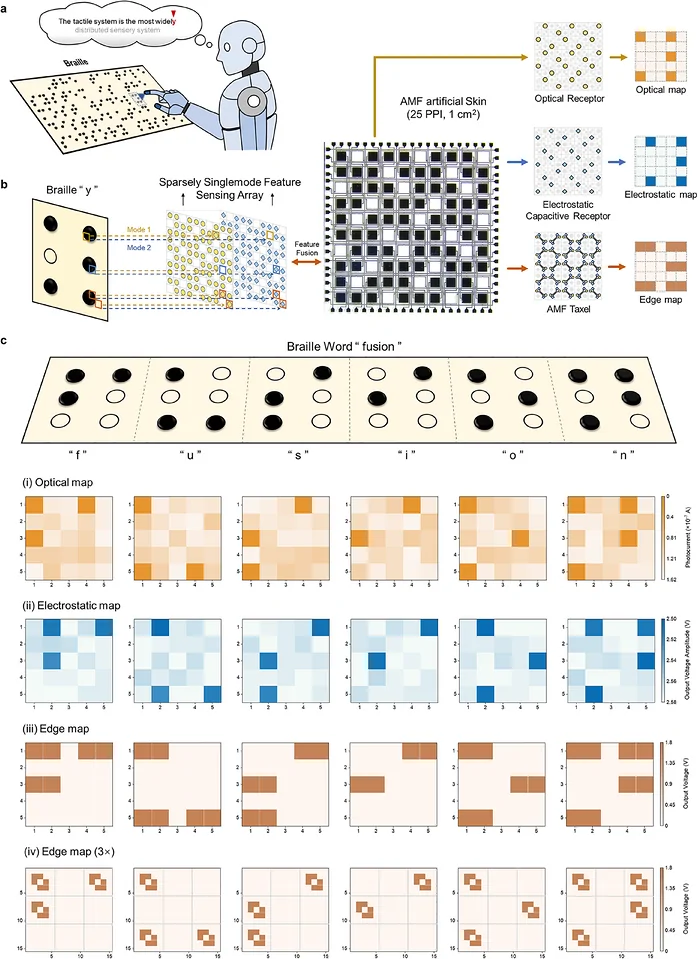
Braille perception based on AMF artificial skin. (a) Schematic of Braille perception of an embodied intelligent robot based on AMF artificial skin. (b) Schematic flowchart of Braille perception based on AMF artificial skin. 25 optical receptors, 25 electrostatic receptors and 50 AMF taxels capture Braille feature into 3 feature maps via multimode pathways. (c) Measured optical maps (i), electrostatic maps (ii), edge maps (iii) and magnified edge maps (3×) (iv) of the Braille word “fusion” captured by AMF artificial skin. Each raised dot covers a 2 × 2 pixel area in (i–iii), and a 4 × 4 pixel area in (iv). Raised dots induce electrostatic stimulation (1 nF), while other areas produce optical stimulation (380 nm, 50 mW, 1 s).

Based on the proposed receptors and CPO, we fabricated an AMF artificial skin sample integrating 1T optical receptors, 1T‐1C electrostatic capacitive receptors, and 2T‐1C AMF taxels. These receptors and taxels form a periodically arrayed structure with 25 PPI resolution over an area of approximately 1 cm^2^. Inspired by the organization of human skin, where individual receptors are sparse and non‐overlapping but distinct receptors partially overlap, the AMF artificial skin incorporates optical and electrostatic capacitive receptors that are each sparsely distributed across 75% of the area, with a 50% overlap between them. The interconnected receptors in these overlapping regions form 50 AMF taxels. Figure S16 shows an optical microscope image and the equivalent circuit of the proposed AMF skin. This customized array topology provides three tactile perception pathways: optical (25%), electrostatic (25%), and their fusion (50%), endowing the skin with comprehensive sensing capabilities with minimal hardware complexity. To experimentally validate the functions of the integrated receptors and AMF taxels within the skin, we conducted pixel‐by‐pixel tactile tests on a probe station using Braille, a globally used symbolic system encoding textual information through densely arranged raised dots (Figure 4a). The test platform for the AMF artificial skin is shown in Figure S17.

The experimental setup and measurement results are presented in Figures S18–S20. The optical and electrostatic capacitive receptors within the array exhibit good device‐to‐device (D2D) reliability, with relative standard deviations (RSD) of both responses below 15% (Figure S21). The active array architecture provides precise electrical isolation at each sensing pixel, enabling selective addressing and readout without crosstalk from neighboring elements, which ensures accurate signal retrieval in large‐scale integration and preserves the fidelity of the D2D responses. As illustrated in Figure 4b, the Braille surface shape is captured via both optical and electrostatic pathways, with in‐situ edge extraction performed by AMF taxels in designated regions. The Braille is down sampled into three patterns: an optical map, an electrostatic map, and an edge feature map. Figure 4c shows the Braille word “fusion” and the corresponding feature maps extracted by the artificial skin, where each raised dot occupies a 2 × 2 pixel area. In the optical maps (Figure 4c (i)), pixels corresponding to the raised dots exhibit near‐zero photocurrent due to the minimal optical stimulation. In the electrostatic maps (Figure 4c (ii)), pixels corresponding to the raised dots exhibit lower output voltage caused by higher electrostatic stimulation. The edge maps (Figure 4c (iii)) highlight the boundaries of the raised dots with large output voltages. This multimodal fusion process achieves an overall information compression ratio of approximately 3.76, effectively reducing the sampled data volume while preserving key features (Figure S22). We also measured larger (3×) “fusion” patterns, where each dot covers a 4×4 pixel area. After 9 repeated experiments (Figure S23), the Braille edges were extracted and demonstrated with finer detail in Figure 4c (iv).

### 2.4 High Tactile Robustness Under Environmental Interference

**FIGURE 5:**
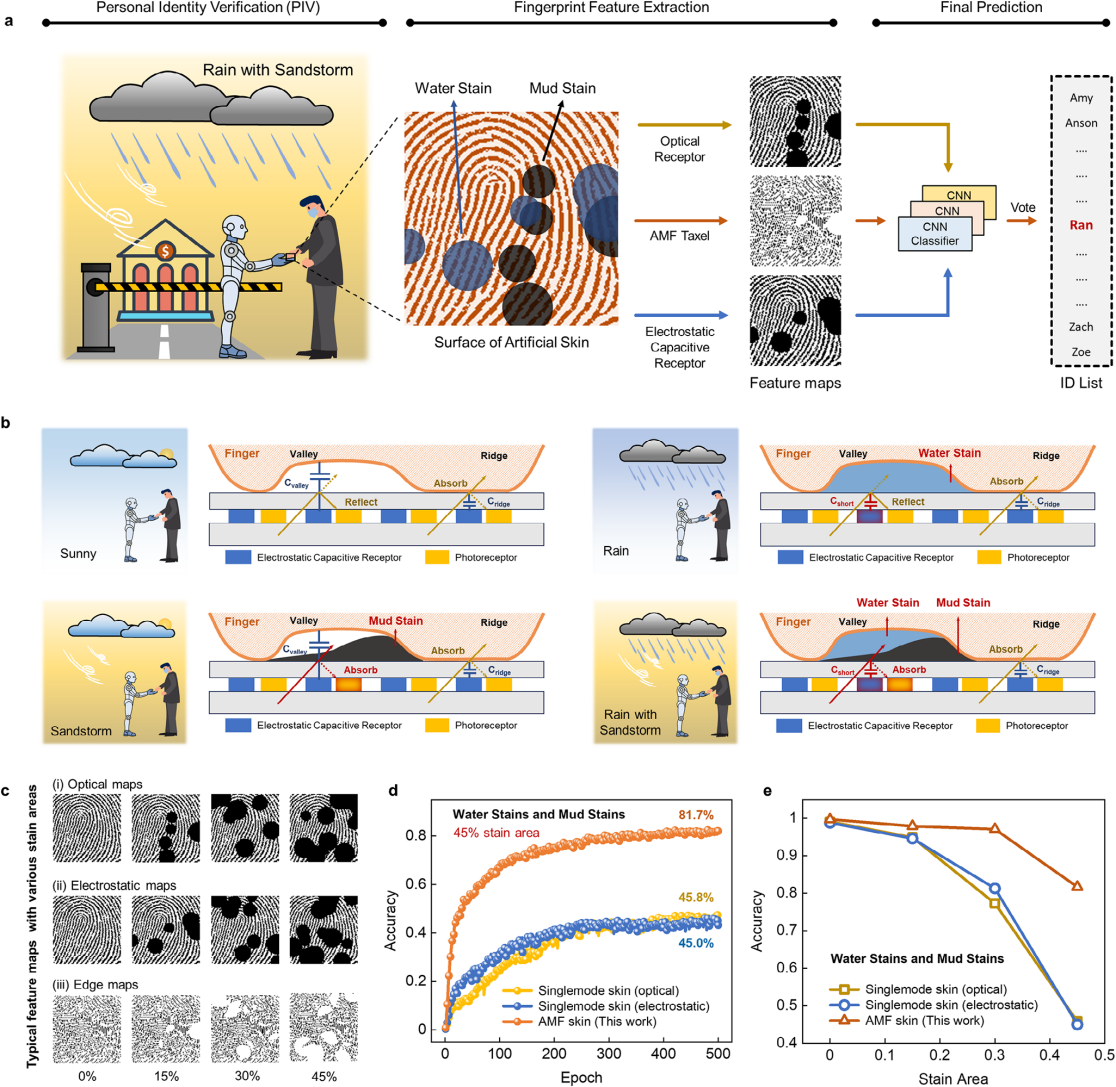
Highly robust PIV process based on AMF artificial skin under environmental interference. (a) Schematic of the fingerprint‐based PIV process using AMF artificial skin under combined rain and sandstorm conditions. b) Mechanism of fingerprint perception by AMF artificial skin and the receptor failure caused by rain or sandstorm. (c) Typical fingerprint optical maps (i), electrostatic maps (ii) and edge maps (iii) extracted by AMF artificial skin with varying water and mud stain areas (0%, 15%, 30%, and 45%) under combined rain and sandstorm conditions. Each feature map measures 96 × 96 pixels. (d) PIV accuracy over training epochs based on different artificial skins with 45% contamination area. (e) PIV accuracy of different artificial skins across various contamination areas (0%, 15%, 30%, and 45%).

As a surface interface, artificial skin is exposed to the environment and subject to various disturbances. The tactile robustness of the AMF artificial skin to such disturbances was evaluated through a simulated fingerprint‐based PIV task under harsh conditions, which is highly sensitive to data integrity. During the simulation, the device behavior of the simulated artificial skin is consistent with that of our experimentally measured 10 × 10 artificial skin. The PIV process is shown in Figure 5a. The AMF artificial skin extracted human fingerprint into three feature maps, which were subsequently fed into customized convolutional neural network (CNN) classifiers (Figure S24) for fusion and personal identification. Environmental contaminants such as rainwater and dust disturbed the PIV process by depositing water and mud onto the artificial skin, disrupting both optical and electrostatic sensing (Figure 5b). Generally, ridges in a fingerprint act on electrostatic capacitive receptors, generating high touch capacitance (C_ridge_) and suppressing optical stimulation by absorbing light. In contrast, valleys produce negligible capacitance (C_valley_) and reflect light to activate the optical receptors. In practice, water stains on the skin often disrupt electrostatic sensing by short‐circuiting the valleys to the skin surface, producing touch capacitance (C_short_) comparable to that of the ridges. Mud stains impair optical sensing by absorbing light, thereby preventing valley activation. These contaminants cause information loss and compromise data integrity.

We established a dataset containing 4000 raw fingerprints (see “Methods” for details) (Figure s25). The dataset was perceived by the AMF artificial skin under combined rain and sandstorm conditions, with typical results shown in Figure 5c. Information loss increased with the severity of contamination. Notably, the edge maps exhibit substantially less information loss compared to the other maps, owing to the AMF taxels' ability to suppress the singlemode disturbances. For comparison, conventional singlemode artificial skins were also considered in Figure S26, which showed perceptual information loss in individual sensing modalities. Figure 5d presents the training results of CNN classifiers with the AMF or conventional artificial skins under both rain and sandstorm conditions. With 45% contamination, the AMF artificial skin achieves an accuracy of 81.7% after 500 training epochs, nearly twice that of the singlemode artificial skin (∼45%). The AMF artificial skin exhibits superior tactile robustness across an environmental contamination range of 0% to 45%, outperforming its singlemode counterparts (Figure 5e). This advantage arises from its multimode sensing and cross‐modal feature extraction capability. Moreover, under isolated rainy or sandstorm conditions, the AMF skin resists singlemode interference and consistently maintains a high recognition accuracy of ∼99% (Figures S27 and S28).

### 2.5 Dynamic‐Taxel‐Density Tactile System

**FIGURE 6:**
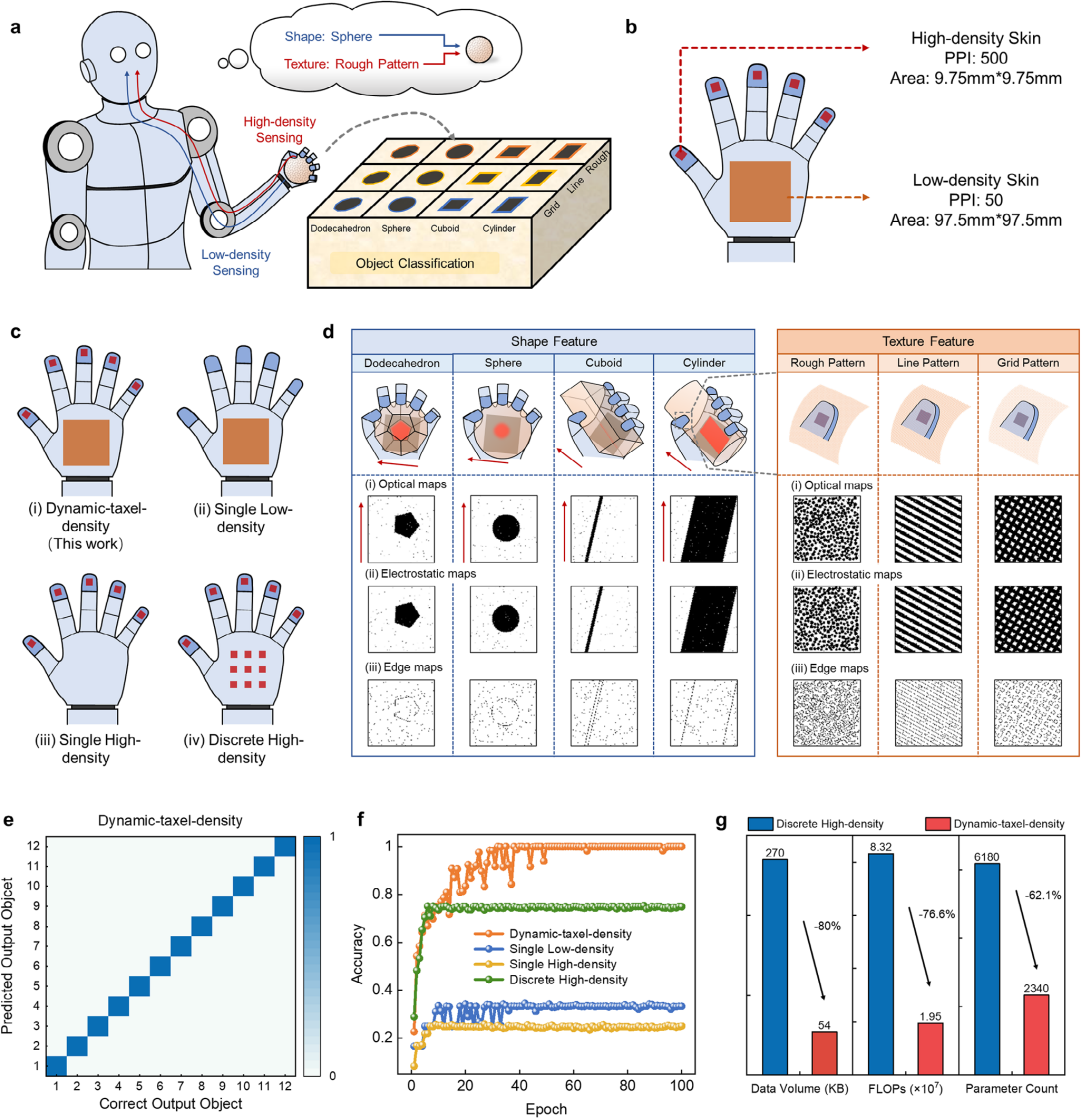
Low‐cost object classification based on dynamic‐taxel‐density tactile system. (a) Schematic of the object classification process using the dynamic‐taxel‐density tactile system for a dexterous hand. (b) Schematic of the dynamic‐taxel‐density tactile system applied to the dexterous hand. (c) Schematic of four tactile systems for the dexterous hand: dynamic‐taxel‐density (i), single low‐density (ii), single high‐density (iii) and discrete high‐ density (iv). (d) Typical shape and texture optical maps (i), electrostatic maps (ii) and edge maps (iii) extracted by the dynamic‐taxel‐density tactile system. Shape features are extracted by the low‐density skin, while texture features are from the high‐density skin. Each feature map is 96 × 96 pixels. (e) Confusion matrix of predicted output versus correct output after 100 training epochs using the dynamic‐taxel‐density tactile system. (f) Classification accuracy across training epochs based on different tactile systems. (g) Comparison of data volume, FLOPs and CNN parameter count between the dynamic‐taxel‐density tactile system and the discrete high‐density tactile system.

As a whole‐body sensing interface, artificial skin generates massive tactile data through numerous embedded receptors, which poses significant challenges for computing power and energy efficiency. Here, we report a simulated tactile system for dexterous hand with dynamic receptor density and introduce a texture‐shape‐dependent object classification task (Figure 6a) to comprehensively evaluate the cost of the tactile system. The system incorporates high‐density skin at the fingertips (500 PPI) and low‐density skin at the palm (50 PPI), resembling the structure of the human hand (Figure 6b). For comparison, three common tactile systems are also introduced (Figure 6c). We constructed an object dataset (see “Methods” for details) with two categories of labels: shape (dodecahedron, sphere, cuboid, and cylinder) and texture (rough, lined, and grid patterns) (Figure S29). The objects were gripped by the dexterous hands, and Figure 6d shows typical feature maps extracted by our tactile system. Additional results from other systems are shown in Figure S30. These maps were then fed into CNN classifiers (Figure S31) for feature fusion, training, and testing. Figure 6e and Figure S32 are the recognition accuracies after 100 training epochs, and our system achieves 100% recognition accuracy. The training details are compared in Figure 6f. The single high‐density or low‐density systems missed either shape or texture information. The discrete high‐density system captured several shape‐related feature maps but failed to reconstruct the complete shape, resulting in misclassification of shape‐similar objects such as the dodecahedron and the sphere in our dataset. Furthermore, the dynamic‐taxel‐density tactile system also possesses more complex capabilities for dynamic object recognition (Figure S33). As the dexterous hand transitions from lightly gripping to firmly gripping an object, the dynamic‐taxel‐density tactile system can determine the object's hardness by identifying whether the extracted shape feature maps change, thereby enabling complex object perception in dynamic scenarios.

We evaluated hardware overhead across data movement, computational cost, and recognition algorithm scale, measured by data volume, floating‐point operations (FLOPs), and CNN parameter count, respectively. The comparison focuses on the dynamic‐taxel‐density system and the discrete high‐density system, both of which are capable of accurate object recognition (Figure 6g and Table S2). Per sensing instance, the dynamic‐taxel‐density system reduces the tactile data movement, computational cost, and recognition algorithm scale by 80%, 76.6%, and 62.1%, respectively. These reductions are primarily achieved by limiting high‐density skin coverage to the fingertips and avoiding redundant high‐precision feature extraction from the palm. As a result, the dynamic‐taxel‐density system achieves comparable perceptual performance with significantly lower computational and hardware cost. In addition, compared to conventional singlemode artificial skins, the AMF artificial skin also reduces data movement by 25% and computational cost by 23.8% (Table S3). These advantages position the AMF‐based dynamic‐taxel‐density tactile system as a promising solution for high‐accuracy, low‐cost full‐body tactile sensing in embodied intelligent robots.

## 3 Discussion

We have reported a bioinspired AMF taxel based on standard TFT platform. This taxel realizes active multimode perception by integrating a 1T optical receptor and a 1T‐1C electrostatic capacitive receptor and employs a specialized arrangement to fuse their outputs for cross‐modal edge extraction. A 10 × 10 AMF artificial skin sample integrating within a 1 cm^2^ area was fabricated, and its tactile function was experimentally validated through a Braille perception task. The artificial skin exhibited excellent tactile robustness, maintaining 81.7% accuracy in fingerprint recognition even under 45% information loss. A simulated dynamic‐taxel‐density tactile system integrated with a dexterous hand was developed, achieving texture‐shape‐dependent object recognition with 100% accuracy, along with 80% less data movement, 76.6% less computational cost, and 62.1% less recognition algorithm scale. Overall, this TFT‐based AMF taxel, achieving robust multimode sensing and early‐stage information processing, demonstrates strong potential to serve as the fundamental units of next‐generation tactile systems. Besides, the TFT platform offers excellent process stability over meter‐scale areas and scalable devices spanning micrometers to nanometers, while the proposed AMF taxel features good D2D uniformity, TFT‐compatible materials, and a simple six‐step fabrication process, enabling low‐cost, large‐area production. The synergy between the AMF taxel and the TFT manufacturing ecosystem offers a promising pathway toward full‐body‐wrapped tactile system featuring dynamic receptor density and low computational cost, enhancing their interaction with the physical world in the future.

## 4 Methods

### 4.1 Design and Fabrication of AMF Taxel

The fabrication of the AMF taxel involves a five‐mask photolithographic process. All patterning is achieved through photolithography and lift‐off processes. First, an 80‐nm aluminum (Al) layer is deposited on a glass substrate via radio‐frequency (RF) magnetron sputtering to serve as the gate electrodes of both the sensing and gating TFTs. Second, a 10‐nm Al_2_O_3_ layer and a 25‐nm ZnO layer are sequentially deposited as the gate dielectric and active layers at 120°C using atomic layer deposition (ALD). Third, after patterning the source/drain (S/D) electrodes, the exposed active region is treated with oxygen plasma (90 W, 90 s) to optimize the interface. Then, another 80‐nm Al layer is deposited via RF magnetron sputtering to form the S/D electrodes of the TFTs and the bottom electrode of the capacitor. Fourth, a 30‐nm Al_2_O_3_ layer is deposited by ALD at 120°C to serve as the capacitor dielectric. Fifth, a 30‐nm Al_2_O_3_ layer is deposited by evaporation on top of the gating transistor channel as a passivation layer. Then, an 80‐nm Al layer is deposited via RF magnetron sputtering above the gating transistor channel as a light‐blocking layer to suppress photosensitivity. Finally, all fabricated devices are annealed at 100°C in ambient air for 1 h to enhance stability. For the sensing transistor, the channel width/length (W/L) ratio is set to 30/10 µm, while for the gating transistor, the W/L ratio is 75/5 µm. The area of the bottom electrode of the capacitor is 1 mm^2^.

### 4.2 Fingerprint‐Based PIV Stimulation

We conducted fingerprint recognition experiments to compare the performance of the AMF artificial skin and singlemode artificial skins. Customized fingerprint datasets were established for both training and testing. For the AMF artificial skin, fingerprints were captured using a 192 × 192 array, from which three 96 × 96 feature maps were extracted. For singlemode artificial skins (optical or electrostatic capacitive), fingerprints were similarly acquired using a 192 × 192 array, yielding a single 192 × 192 feature map. The pixels in the feature maps had binary values (1 or 0). Accordingly, three datasets were constructed for training and testing of CNNs. For the AMF artificial skin, the three feature maps were separately fed into three identical CNN classifiers, and the final classification result was obtained by fusing their outputs via a soft voting strategy, assigning equal weight to each prediction. For the singlemode artificial skins, the feature maps were directly input into a single CNN classifier. Standard backpropagation algorithms were used for network training and testing. The training dataset consisted of 4000 fingerprint classes, with 30 samples per class. The testing dataset contained the same 4000 classes, with 10 samples per class, and all test samples were independent from those used in training.

### 4.3 Object Recognition Stimulation

To evaluate the performance of the dynamic‐taxel‐density tactile system, we conducted object recognition experiments using a customized dataset that incorporated both shape and texture features. All tactile systems used AMF artificial skin with a resolution of 192 × 192 (different PPI), which down sampled object characteristics into three 96 × 96 feature maps: optical, electrostatic, and edge. For each object, the dynamic‐taxel‐density system extracted 3 × 2 feature maps (three shape‐related and three texture‐related); the single density systems (either high‐ or low‐density) extracted 3 × 1 feature maps (containing either shape or texture only); the fully discrete high‐density system extracted 3 × 1 × 9 shape‐related feature maps and 3 × 1 texture‐related maps. To simulate real‐world signal variability, 2% Gaussian noise was added to each feature map. Based on these settings, four datasets were constructed for CNN training and testing. Across all systems, optical, electrostatic, and edge feature maps were independently processed by identical CNN classifiers. Their outputs were then combined via soft voting with equal weights. For the dynamic‐taxel‐density and fully discrete high‐density systems, dual‐branch parallel CNN architectures were employed, where texture and shape feature maps (single‐channel or nine‐channel) were respectively fed into separate branches. For the single density systems, normal single‐branch CNN architectures were employed. Standard backpropagation algorithms were used for network training and testing. The training dataset consisted of 12 object categories, with 300 samples per class. The testing dataset contained the same 12 object categories, with 100 samples per class, and all test samples were independent from those used in training.

## Author Contributions

**Sihao Wu**: methodology, validation, visualization, software, data curation, formal analysis, writing – original draft, writing – review and editing, investigation. **Tingchen Yi**: validation, visualization, data curation. **Chuanlin Sun**: validation, visualization. **Lutong Wang**: data curation, validation, visualization, formal analysis. **Aoran Xu**: software, formal analysis, data curation, visualization. **Zheng Zhou**: conceptualization, supervision, project administration, resources, funding acquisition, writing – original draft, writing – review and editing. **Haotong Zhu**: software, methodology. **Lifeng Liu**: supervision, resources, funding acquisition. **Li Zhou**: supervision, resources. **Jiaqi Li**: data curation, formal analysis. **Yimao Cai**: supervision, resources, funding acquisition. **Dedong Han**: supervision, resources, funding acquisition. **Junchen Dong**: validation, visualization, supervision, resources, funding acquisition. **Xing Zhang**: funding acquisition, resources, supervision.

## Funding

This work is supported by the Beijing Nova Program (20230484256 and 20240484536), the National Key R&D Program of China (2023YFB3611600).

## Conflicts of Interest

The authors declare no conflicts of interest.

## Supporting information




**Supporting File**: advs76190‐sup‐0001‐SuppMat.docx

## Data Availability

The data that support the findings of this study are available from the corresponding author upon reasonable request.
