# Supplementary material for: Thin‐Film Transistor Based Active Taxel for Multimode Tactile Perception and Fused Processing
Source: Adv Sci (Weinh). 2026 Jun 17:e76190. Online ahead of print. doi: 10.1002/advs.76190 (PMC13336976; doi:10.1002/advs.76190)
Supplement: Supplementary file 1 — Supporting File: advs76190‐sup‐0001‐SuppMat.docx [file ADVS-9999-e76190-s001.docx]

Supporting Information

**Thin-Film Transistor based Active Taxel for Multimode Tactile Perception and Fused Processing**

*Sihao Wu, Zheng Zhou^*^, Aoran Xu, Lutong Wang, Tingchen Yi, Chuanlin Sun, Haotong Zhu, Jiaqi Li, Junchen Dong^*^, Li Zhou, Lifeng Liu, Yimao Cai, Dedong Han, Xing Zhang^*^*

**This PDF file includes:**

Note S1

Figure S1 to S33

Table S1 to S3

References

**Note S1. Photo-response mechanism of ZnO TFTs**

Upon irradiation by a 380 nm laser, photogenerated carriers in the ZnO TFTs are primarily generated through two mechanisms: intrinsic excitation of the ZnO film, and ionization of V_o_ described by the following reactions:

$$V_{o}\underset{\to}{hv}V_{o}^{2+}+{2e}^{-} (1)$$

where $hv$ is a photon and $e^{-}$ is the free electron. These photogenerated carriers are driven into the channel by the electric field, significantly increasing the channel's carrier concentration.

We take the ionization process of V_o_ as an example to derive the photo-response mechanism. Firstly, the distribution of V_o_ follows an exponential form:^[1,2]^

$$n_{t}\left( E \right)=\frac{N_{t}(0)}{kT_{0}}\exp\left( \frac{E_{h}-E}{kT_{0}} \right) (2)$$

where $n_{t}\left( E \right)$ is the density of V_o_ per unit energy, $N_{t}(0)$ is the total V_o_ concentration, $E_{h}$ is the highest energy barrier required for electrons to be excited from V_o_, k is Boltzmann constant, and $T_{0}$ is the characteristic temperature related to the materials and the width of V_o_ distribution. The ionization rate of V_o_ under irradiation depends on the initial energy level and follows:

$$R\left( E \right)=R_{0}\exp\left( \frac{E}{kT} \right) (3)$$

where $R_{0}$ is a constant related to the optical power (P), and $T$ is the absolute temperature. Due to the high energy barrier for V_o_^2+^ to capture electrons and revert to V_o_,^[3]^ the recombination process can be neglected. Accordingly, the rate of electron generation over time is given by:

$$\frac{dN(t)}{dt}=\int_{E_{l}}^{E_{h}} 2*n_{t}\left( E \right)R\left( E \right)dE (4)$$

The lower bound $E_{l}$ corresponds to the minimum observable ionization energy during measurement and is given by:

$$E_{l}=kTln\left( \nu_{0}t \right) (5)$$

where $\nu_{0}$ is the attempt-to-escape frequency, typically approximated by the characteristic lattice vibration frequency (~$\mathrm{kT}/h$).^[4]^ By solving equations (3)-(6), the time-dependent carrier concentration is:

$$N\left( t \right)={2N}_{t}\left( 0 \right)\left\{ 1-exp\left[ {-\left( \frac{t}{\tau} \right)}^{\beta} \right] \right\} (6)$$

with parameters:

$$\tau=\frac{1}{\nu_{0}}exp\left( \frac{E_{\tau}}{kT} \right) (7)$$

$$E_{\tau}=kT_{0}ln\left[ \frac{\nu_{0}\left( 1-\beta\right)}{R_{0}} \right]+E_{h} (8)$$

$$\beta=T/T_{0} (9)$$

where $\tau$ is the average ionization time_,_ $E_{\tau}$ is the average activation energy and $\beta$ (0 < $\beta$ ≤ 1) is a constant reflected the distribution of time constants around $\tau$.^[5,6]^ Since the ZnO TFT has a sufficiently long channel and operates under low applied voltage, we assume that electron drift velocity $v$ is linearly proportional to the electric field ($E$):

$$v=\mu_{n}E=\frac{\mu_{n}V_{DS}}{L} (10)$$

where $\mu_{n}$ is the carrier mobility, $V_{\mathrm{DS}}$ is the drain-source voltage, and L is channel length. Given that the carrier concentration in the ZnO film is relatively uniform along the channel and the channel length is sufficiently long, the diffusion current can be neglected. Hence, the increase in channel current over time is:

$$\Delta I\left( t \right)={qvS*2N}_{t}\left( 0 \right)\left\{ 1-exp\left[ {-\left( \frac{t}{\tau} \right)}^{\beta} \right] \right\} (11)$$

Assuming uniform carrier distribution across the ZnO film, the cross-sectional area ($S$) is:

$$S=W*t_{ss} (12)$$

where W is channel width and $t_{\mathrm{ss}}$ is the thickness of ZnO film. Substituting $v$ and $S$ yields:

$$\Delta I\left( t \right)=C\left\{ 1-exp\left[ {-\left( \frac{t}{\tau} \right)}^{\beta} \right] \right\} \left( 13 \right)$$

with

$$C={2N}_{t}\left( 0 \right)*q\mu_{n}t_{ss}V_{DS}\frac{W}{L} \left( 14 \right)$$

Similarly, intrinsic photoexcitation of the ZnO film contributes a current increase that also follows a stretched exponential form. Thus, the total photocurrent under optical stimulation can be expressed as a double stretched exponential equation:

$$I\left( t \right)=I_{0}+C_{1}\left\{ 1-exp\left[ {-\left( \frac{t}{\tau_{1}} \right)}^{\beta_{1}} \right] \right\}+C_{2}\left\{ 1-exp\left[ {-\left( \frac{t}{\tau_{2}} \right)}^{\beta_{2}} \right] \right\} \left( 15 \right)$$

where $I_{0}$ is the baseline current, and the subscripts 1 and 2 denote contributions from V_o_ ionization and ZnO intrinsic excitation, respectively.

After light exposure ceases, the photocurrent decay also follows a double stretched exponential form:

$$I\left( t \right)={I_{0}}^{'}+C_{3}exp\left[ {-\left( \frac{t}{\tau_{3}} \right)}^{\beta_{3}} \right]+C_{4}exp\left[ {-\left( \frac{t}{\tau_{4}} \right)}^{\beta_{4}} \right] \left( 16 \right)$$

where ${I_{0}}^{'}$ is the steady-state current, and the decay terms correspond to the recombination of $V_{o}^{2+}$ and free electrons, and of electrons with holes, respectively.

**
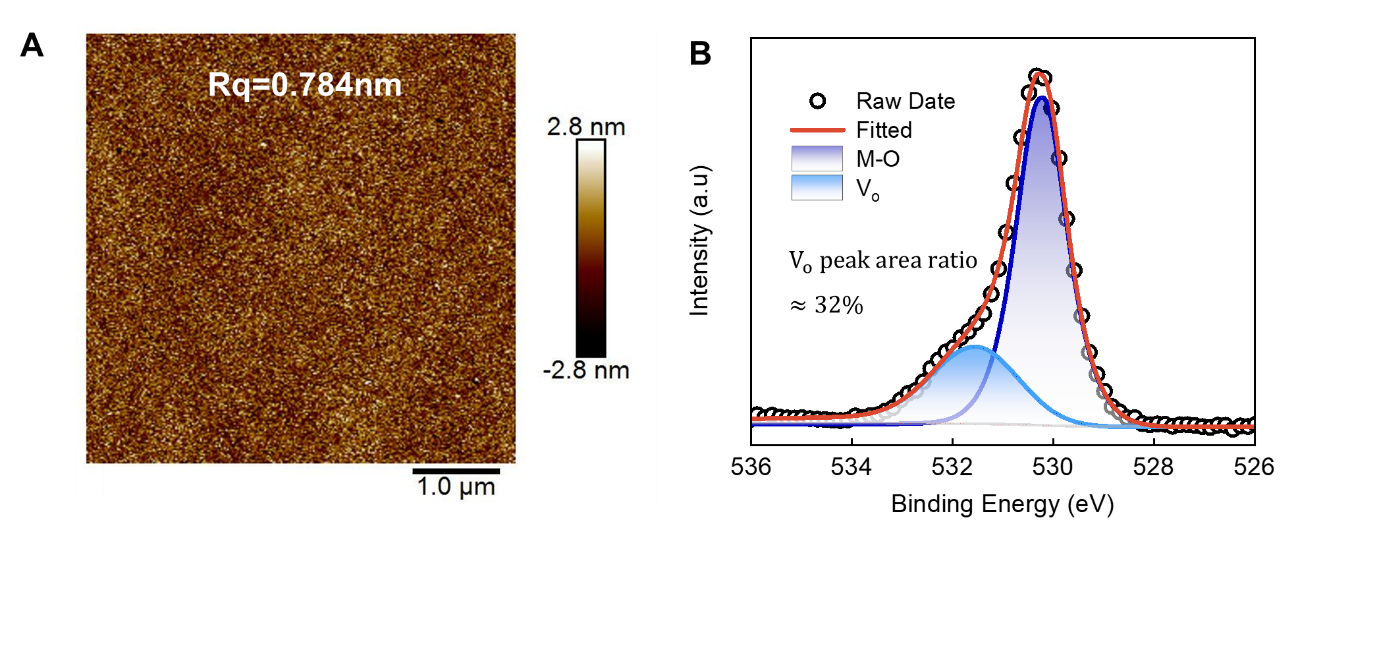
**

**Figure S1. Surface morphology and oxygen vacancy density of ZnO films.** (**A**) AFM image of a ZnO film. (**B**) O 1s spectra of a ZnO film. The ZnO film exhibits low root-mean-square roughness (R_q_) and high oxygen vacancy density.


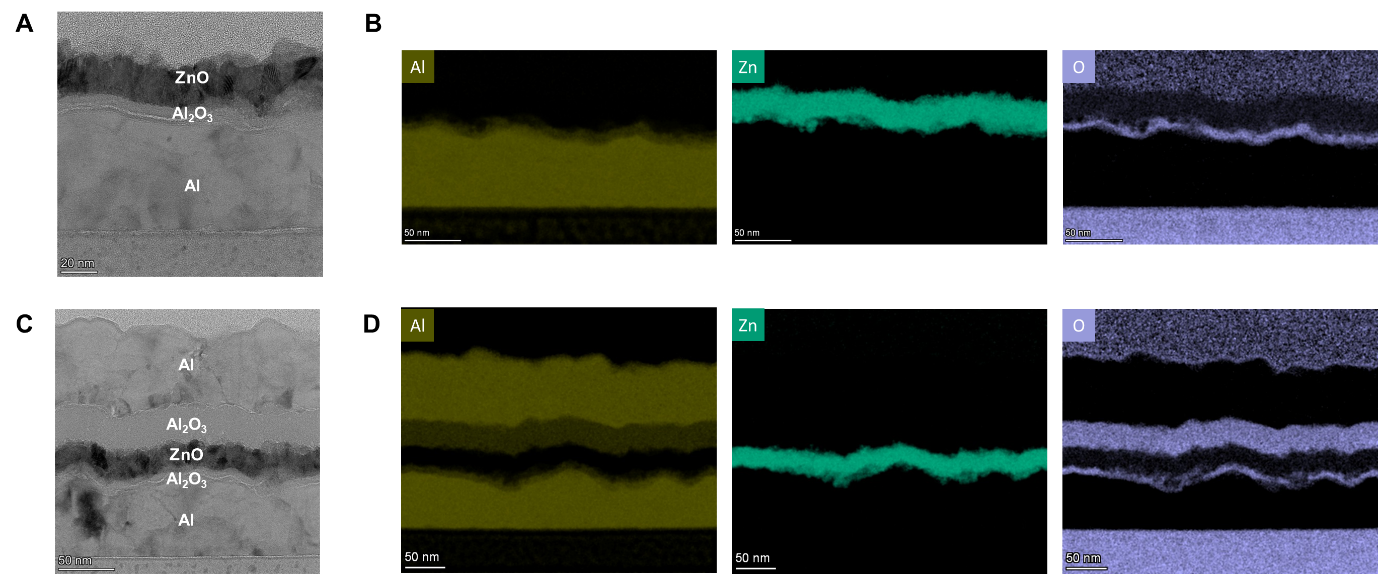


**Figure S2. Cross-sectional structure and elemental distribution of ZnO TFTs.** (**A**) Transmission electron microscope (TEM) image of the intersection of the ZnO TFTs. (**B**) Energy dispersive spectroscopy (EDS) mapping of the intersection of the ZnO TFTs. (**C**) Transmission electron microscope (TEM) image of the intersection of the ZnO TFTs with an added light-blocking layer. (**D**) Energy dispersive spectroscopy (EDS) mapping of the intersection of the ZnO TFTs with an added light-blocking layer. The aluminium deposited by radio-frequency magnetron sputtering exhibits large grain sizes and induces rough surface.

**
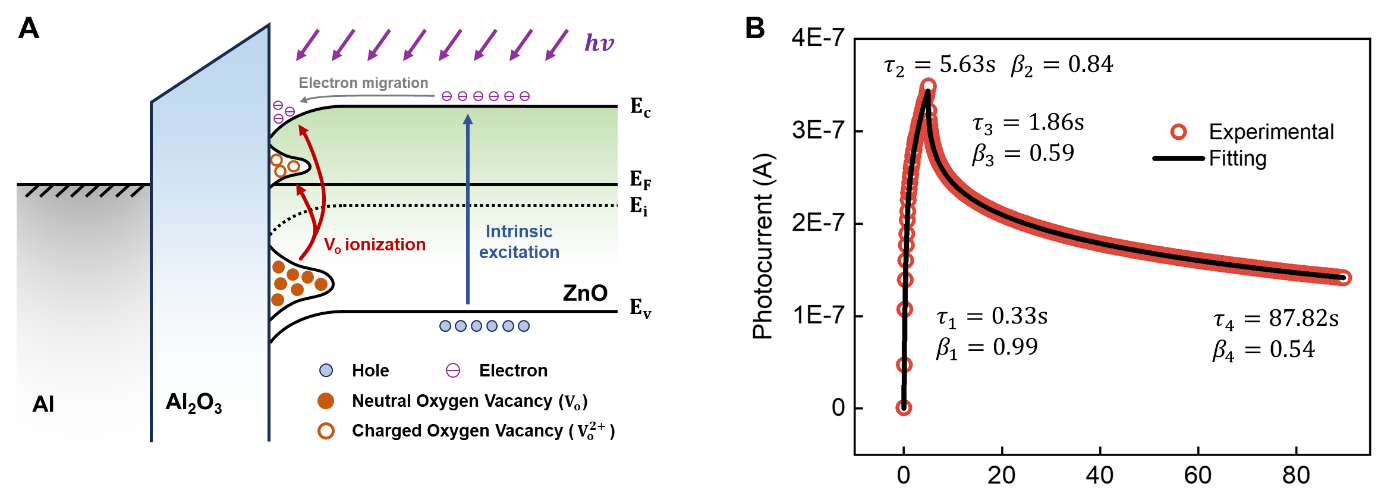
**

**Figure S3. Photo-response mechanism of ZnO TFTs.** (**A**) Schematic of two photo-response mechanisms of ZnO TFTs. (**B**) Time-dependent photocurrent measured under optical stimulation (wavelength of 380 nm, optical power of 5 mW, irradiation time of 5 s) along with model fitting. The excitation and decay processes are respectively fitted using two double stretched exponential models (Note S1).

**
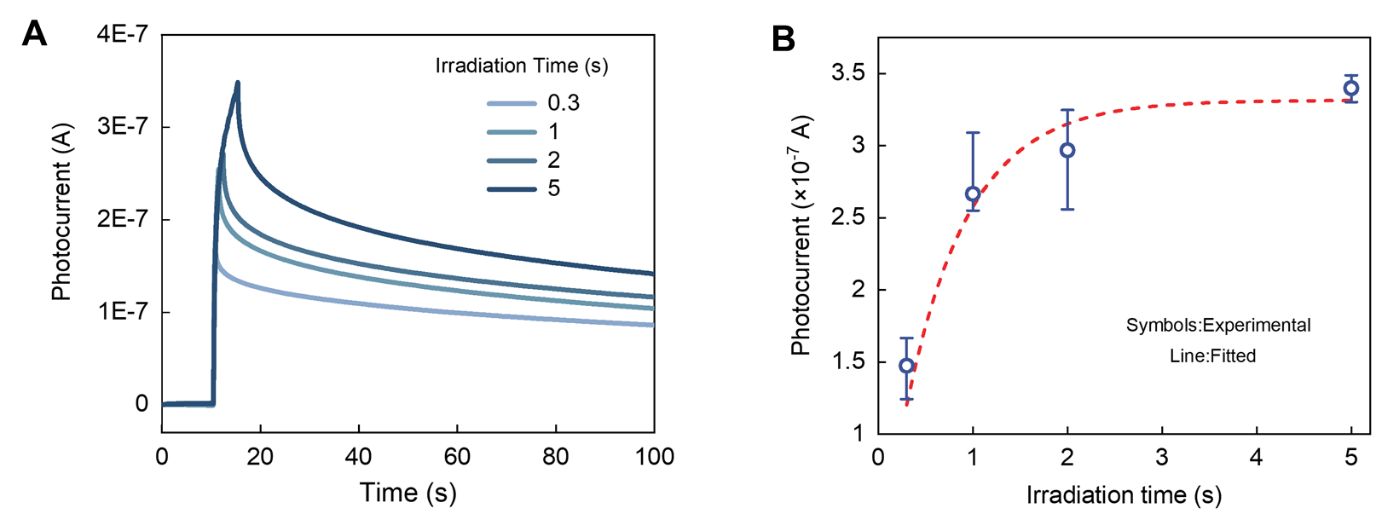
**

**Figure S4. Influence of irradiation time on the photocurrent of optical receptors.** (**A**) Photocurrent of the optical receptor under varying optical stimulation (irradiation time of 0.3, 1, 2, 5 s, optical power of 5 mW). The applied V_gs_ and V_ds_ are 0 V and 0.1 V, respectively. (**B**) Peak photocurrents of optical receptors as a function of irradiation time (0.3-5 s) under 5 mW optical power. The error bars indicate 3 repeated measurements.


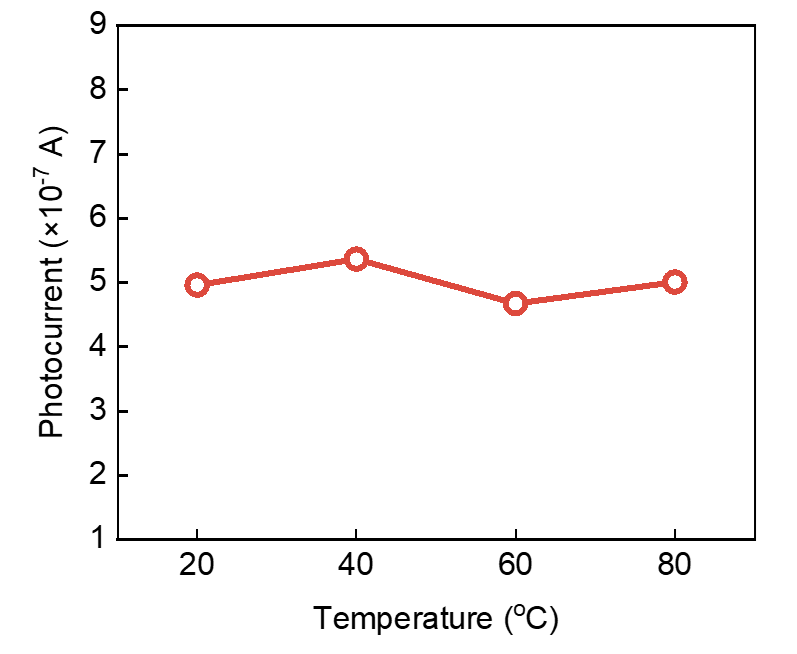


**Figure S5. Typical response of the optical receptor (V_gs_ of 0 V, V_ds_ of 0.1 V, wavelength of 380 nm, optical power of 50 mW, irradiation time of 0.3 s) under varying temperature.** The response RSD of the optical receptor is 5.69%.

**
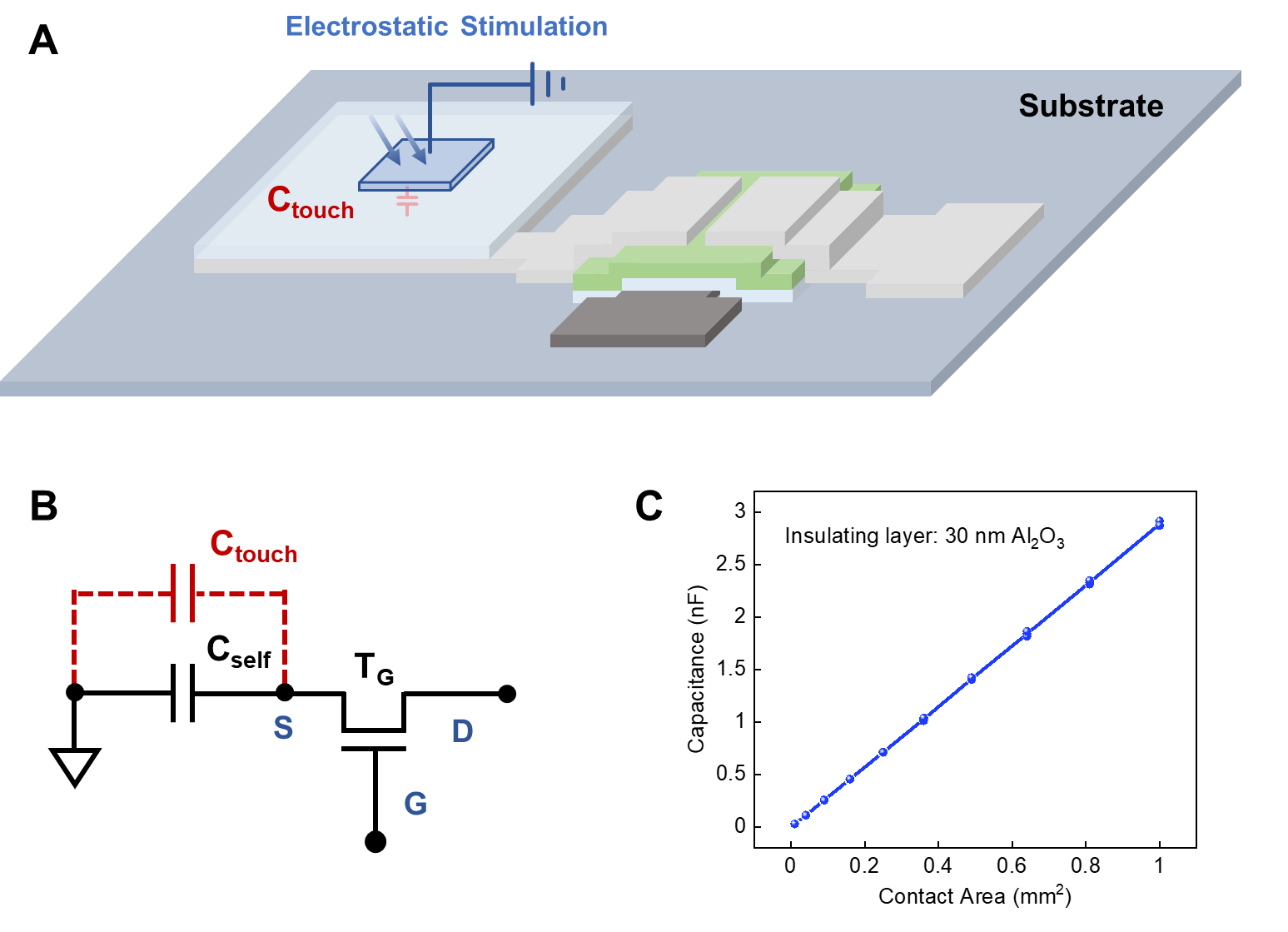
**

**Figure S6. Proportional relationship between C_touch_ and contact area in electrostatic capacitive receptors.** (**A**) Schematic of C_touch_ formation in the electrostatic capacitive receptor. (**B**) Equivalent circuit of the electrostatic capacitive receptor with electrostatic stimulation. Self-capacitance (C_self_) is composed of a series of parasitic capacitances, including source-drain capacitance, gate-source capacitance, and the capacitance formed between the electrode plate and the ground through the substrate. C_touch_ and C_self_ are in parallel. (**C**) Measured capacitance of C_touch_ as a function of contact area. A linear relationship is observed, with an extracted unit-area capacitance of ~287 nF/mm^2^.

**
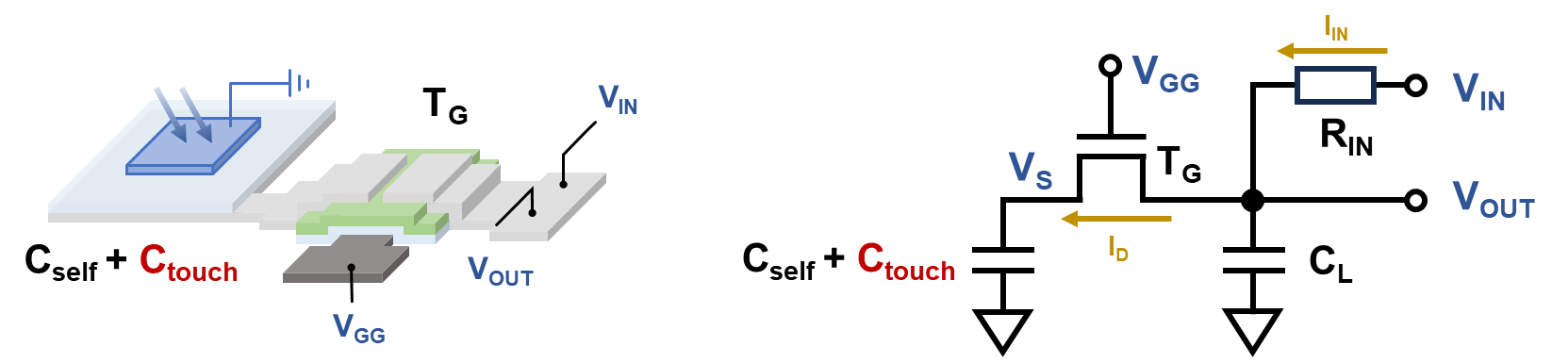
**

**Figure S7. Parallel readout circuit for detecting electrostatic stimulation of the electrostatic capacitive receptor.** The R_IN_ and C_L_ are 25 kΩ and 1 nF, respectively. The input voltage (V_IN_) is a square wave signal with a frequency of 10 kHz, a peak-to-peak voltage of 3 V (average voltage of 0 V) and a duty cycle of 50%. Considering the half-cycle where V_IN_ is 1.5 V, C_L_ is in the charging phase. We simplify the process of the output voltage (V_OUT_) rising with the increase in charging time as a linear process. The coupling relationship between V_IN_ and V_OUT_ can be expressed as:

$$\left[ \frac{\left( V_{IN}-V_{OUT, 0} \right)+\left( V_{IN}-V_{OUT, 1} \right)}{{2R}_{IN}}-\bar{I_{D}} \right]*t=\left( V_{OUT,1}-V_{OUT, 0} \right)*C_{L} (1)$$

where $V_{OUT, 0}$ is $V_{\mathrm{OUT}}$ before charged, $V_{OUT, 1}$ is $V_{\mathrm{OUT}}$ after charged, $\bar{I_{D}}$ is the average channel current during the charging process and t is the half-cycle of $V_{\mathrm{IN}}$. After simplification, the output voltage amplitude ($\Delta V=V_{OUT,1}-V_{OUT, 0}$) can be expressed as:

$$\Delta V=\frac{2\left( V_{IN}-V_{OUT, 0}-\bar{I_{D}}*t*R_{IN} \right)}{{2R}_{IN}*C_{L}*t} (2)$$

According to the expression above, the output voltage amplitude $\Delta V$ exhibits a negative correlation with the average channel current $\bar{I_{D}}$. Further, $\bar{I_{D}}$ can be expressed using the following formulas:

$$\bar{I_{D}}=\mu_{n}C_{ox}\frac{W}{L}\left[ \left( V_{GG}-\bar{V_{S}}-V_{TH} \right)\left( \bar{V_{OUT}}-\bar{V_{S}} \right)-\frac{1}{2}\left( \bar{V_{OUT}}-\bar{V_{S}} \right)^{2} \right] (3)$$

$$\bar{V_{S}}=\frac{\bar{I_{D}}*t}{(C_{self}+C_{touch})} (4)$$

where $\mu_{n}$ is the carrier mobility, $C_{\mathrm{ox}}$ is the gate oxide capacitance, $\frac{W}{L}$ is the width-to-length ratio, $V_{\mathrm{GG}}$ is the gate voltage, $\bar{V_{S}}$ is the average source voltage, $V_{\mathrm{TH}}$ is the threshold voltage and $\bar{V_{\mathrm{OUT}}}$ is the average output voltage. Therefore, both the increase in $C_{\mathrm{touch}}$ and $V_{\mathrm{GG}}$ will lead to an increase in $\bar{I_{D}}$, which in turn causes a decrease in $\Delta V$.

**
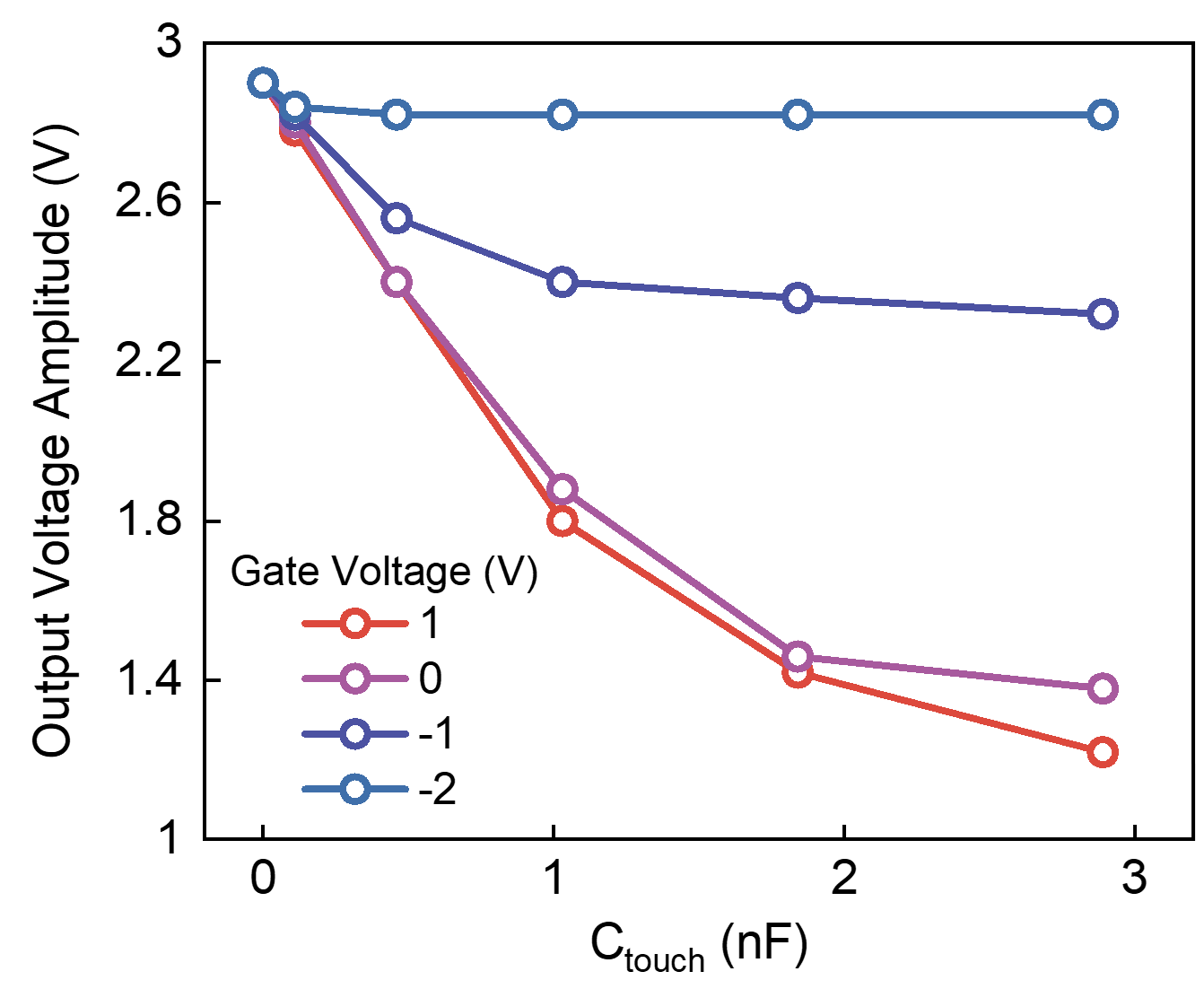
**

**Figure S8. Gate-voltage-dependent modulation of output voltage amplitude in electrostatic capacitive receptors.** The increase in gate voltage effectively broadens the sensing window of the electrostatic capacitive receptor.


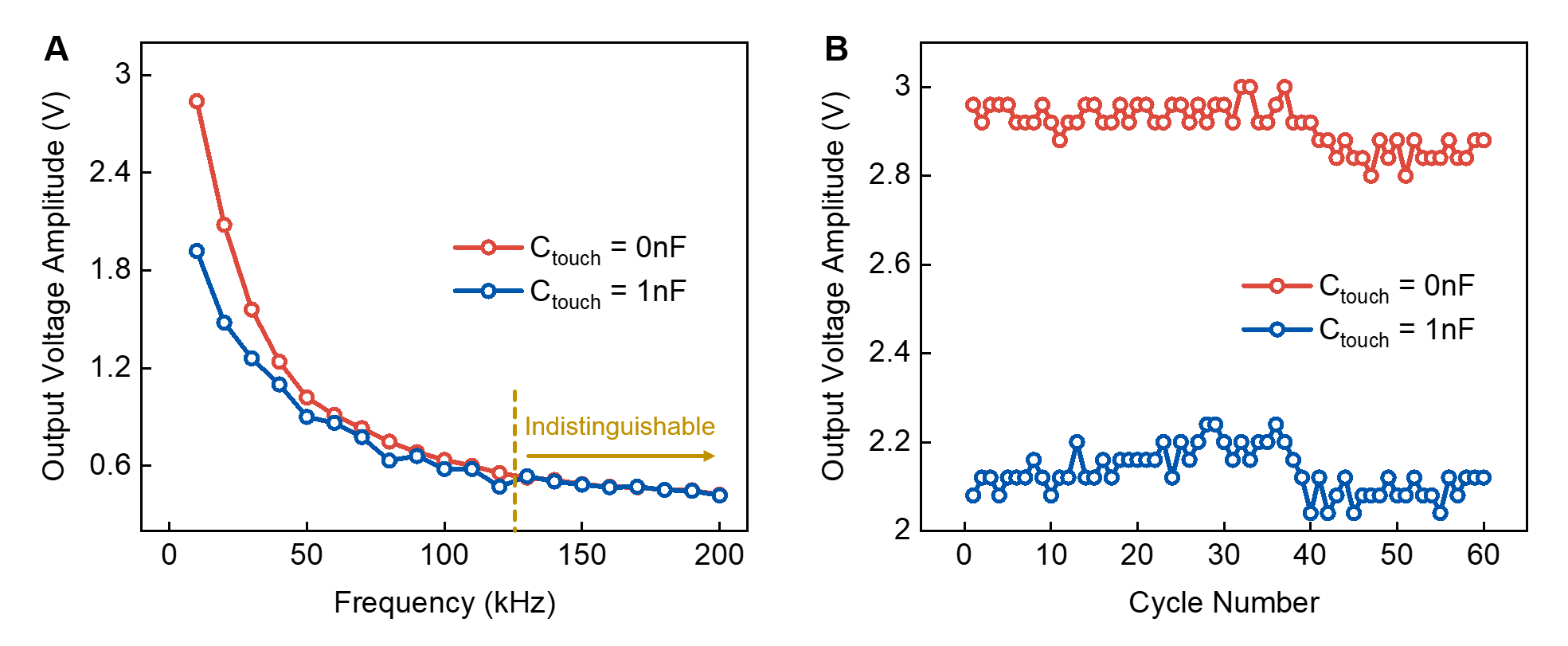


**Figure S9. Response time and cycle-to-cycle (C2C) stability of the electrostatic capacitive receptor.** (**A**) Output voltage amplitude of the electrostatic capacitive receptor under varying operating frequencies (gate voltage of 0 V). When the operating frequency reaches 130 kHz, the electrostatic capacitive receptor is no longer able to distinguish the magnitude of C_touch_, indicating that the cutoff frequency (120 kHz) has been reached. (**B**) Cyclic stability of the electrostatic capacitive receptor (gate voltage of 0 V). Over the 60 measured cycles, the output voltage amplitude relative standard deviation (RSD) remained remarkably low, at 4.93% for C_touch_=0 nF and 5.12% for C_touch_=1 nF.


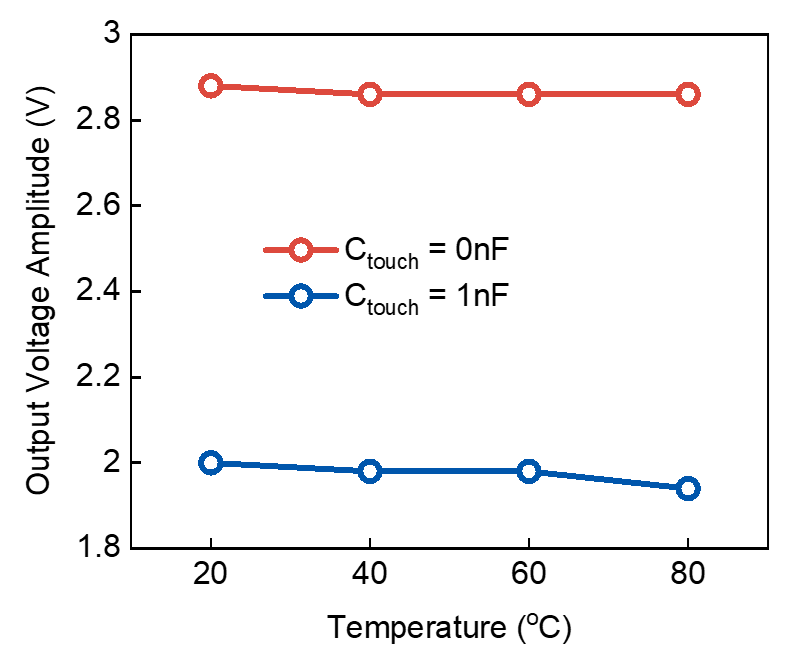


**Figure S10. Typical response of the electrostatic capacitive receptor (V_g_ of 0 V) under varying temperature.** The response RSD of the electrostatic capacitive receptor is 1.27% (C_touch_ = 1 nF).


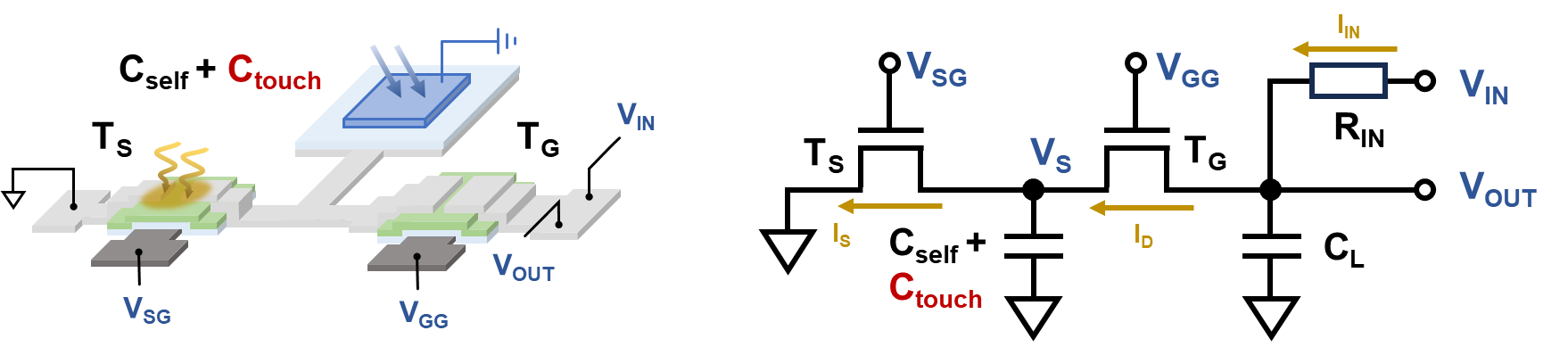


**Figure S11. Parallel readout circuit for dual-stimulation perception of the AMF taxel.** The R_IN_ and C_L_ are 25 kΩ and 1 nF, respectively. The input voltage (V_IN_) is a square wave signal with a frequency of 10 kHz, a peak-to-peak voltage of 3 V (average voltage of 0 V) and a duty cycle of 50%. Considering the half-cycle where V_IN_ is 1.5 V. Similar to the electrostatic capacitive receptor (Figure S7), the expression for the output voltage amplitude is:

$$\Delta V=\frac{2\left( V_{IN}-V_{OUT, 0}-\bar{I_{D}}*t*R_{IN} \right)}{{2R}_{IN}*C_{L}*t} (1)$$

where $V_{OUT, 0}$ is $V_{OUT}$ before charged, $\bar{I_{D}}$ is the average channel current of gating transistor during the charging process and t is the half-cycle of $V_{IN}$. Therefore, $\Delta V$ also exhibits a negative correlation with $\bar{I_{D}}$. Further, $\bar{I_{D}}$ can be expressed using the following formulas:

$$\bar{I_{D}}=\mu_{n,G}C_{ox,G}\frac{W_{G}}{L_{G}}\left[ \left( V_{GG}-\bar{V_{S}}-V_{TH,G} \right)\left( \bar{V_{OUT}}-\bar{V_{S}} \right)-\frac{1}{2}\left( \bar{V_{OUT}}-\bar{V_{S}} \right)^{2} \right] (2)$$

$$\bar{V_{S}}=\frac{(\bar{I_{D}}-\bar{I_{S}})*t}{(C_{S}+C_{touch})} (3)$$

$$I_{S}=I_{0}+k*P (4)$$

where $\mu_{n,G}$, $C_{ox,G}$, $\frac{W_{G}}{L_{G}}$, $V_{GG}$, $\bar{V_{S}}$, $V_{TH,G}$ are the carrier mobility, gate oxide capacitance, width-to-length ratio, gate voltage, average source voltage and threshold voltage of the gating transistor, respectively, $\bar{V_{OUT}}$ is the average output voltage, $\bar{I_{S}}$ is the average channel current of sensing transistor, $I_{0}$ is the initial channel current of sensing transistor, k is a constant coefficient, and P is the optical power. Therefore, higher P and $C_{touch}$ will reduce $\bar{V_{S}}$, thereby increasing $\bar{I_{D}}$ and leading to a decrease in $\Delta V$.


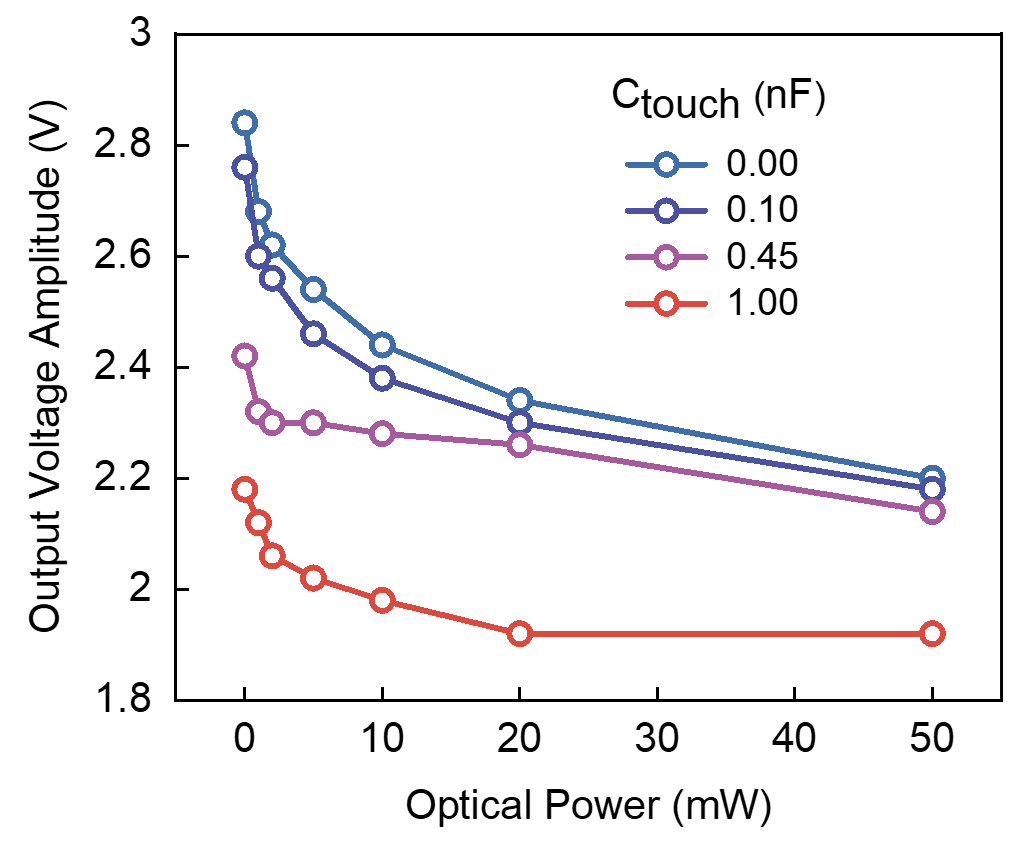


**Figure S12. Output voltage amplitude of the AMF taxel with various optical power and C_touch_.**

**
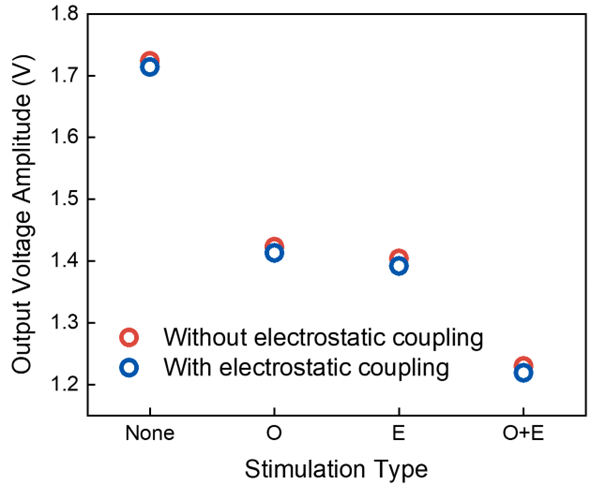
**

**Figure S13. Simulated output of the AMF taxel with and without touch-induced electrostatic coupling.** For the AMF taxel, touch-induced electrostatic coupling primarily occurs at the source, drain, and gate electrodes of the TFT. In our design, the area of the TFT source, drain, and gate electrodes is much smaller than that of the capacitive plates, so the coupled capacitance is only 1/50 of the capacitor. Using Cadence simulation tools, when touch-induced electrostatic coupling is considered, the output decreases by only approximately 0.5% overall, which can be regarded as negligible.


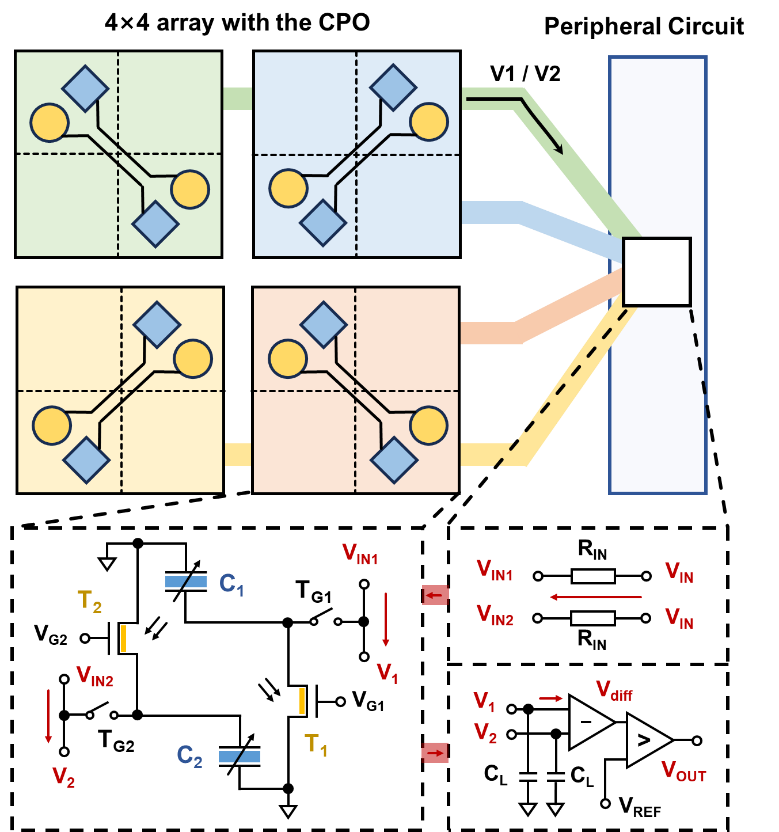


**Figure S14. Equivalent circuit of the cross-modal operator (CPO) with the peripheral circuit.** Firstly, the two AMF taxels of the CPO are sequentially selected for sensing, producing two output signals, V1 and V2. Secondly, V1 and V2 are then fed into a differential amplifier to obtain the differential voltage V_diff_. Finally, V_diff_ is compared with a reference voltage V_REF_ (0.2 V) using a hysteresis comparator, producing a binary output that indicates whether a region is an edge or a non-edge.


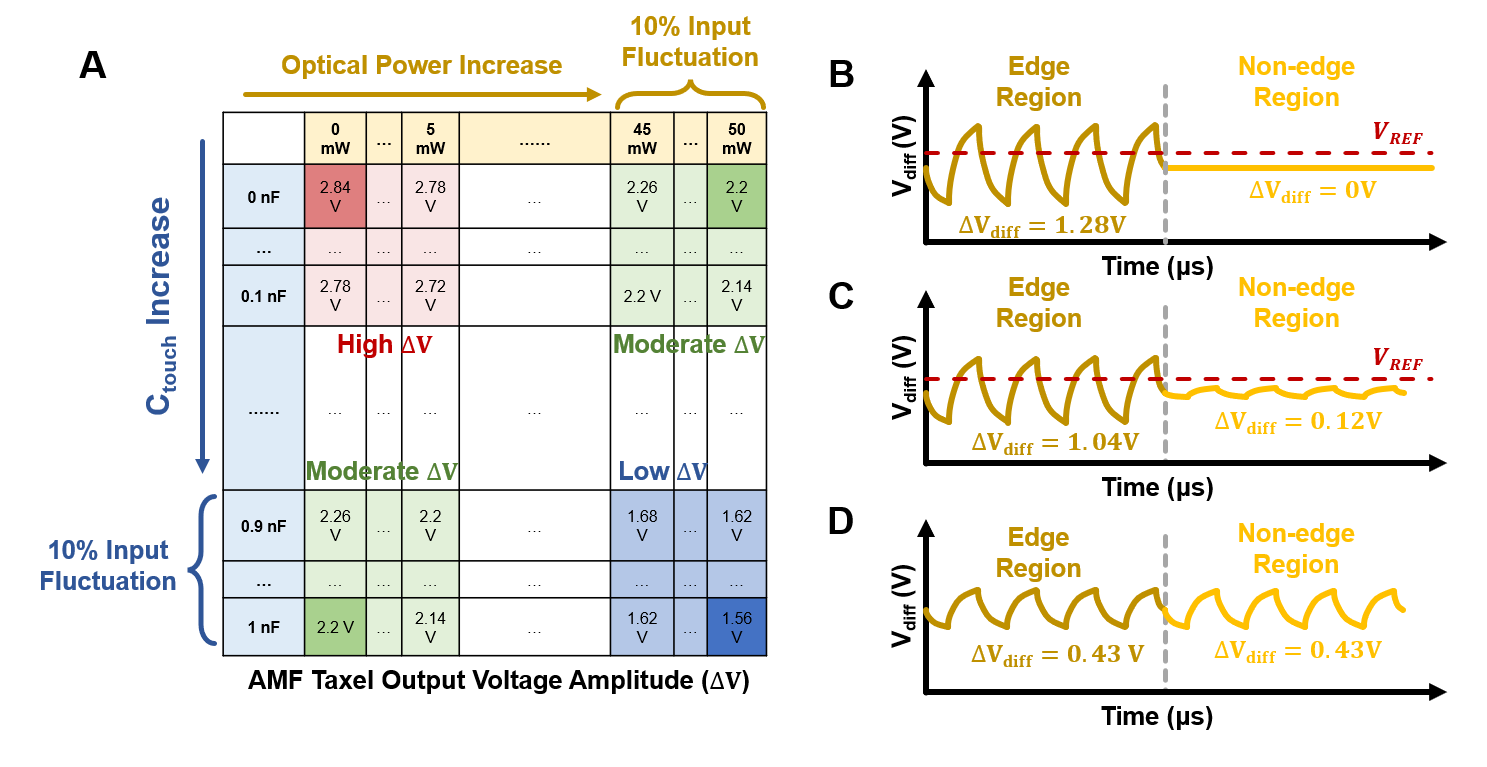


**Figure S15.** (**A**) The output voltage amplitude (∆V) of the AMF taxel under non-ideal multi-value input conditions (10% input fluctuation). The AMF taxel outputs are approximated as varying linearly with either C_touch_ or optical power. Schematic illustration of the CPO output voltage (V_diff_) for edge region and non-edge region under (B) ideal binary inputs, (C) 10% input fluctuation, and (D) 1/3 input fluctuation.

**
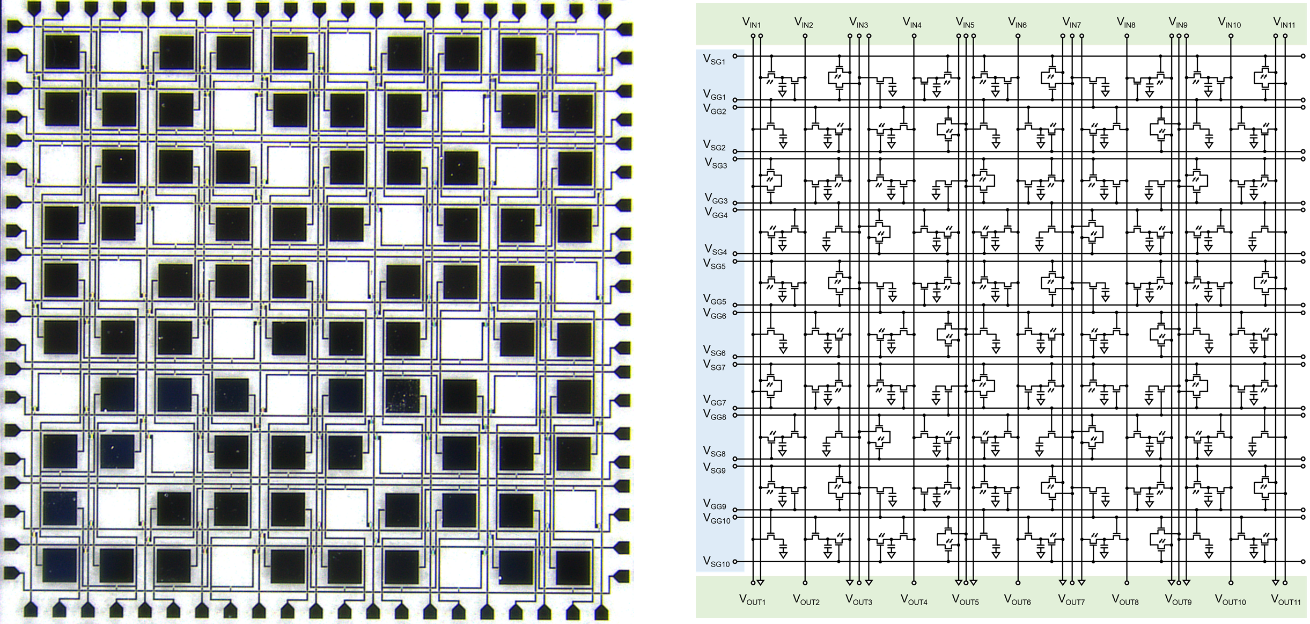
**

**Figure S16. Optical microscope image and equivalent circuit of the AMF artificial skin.** The prototype AMF artificial skin is composed of 75 optical receptors and 75 electrostatic capacitive receptors. Among them, 50 optical and 50 electrostatic capacitive receptors are diagonally or anti-diagonally interconnected to form 50 AMF taxels.


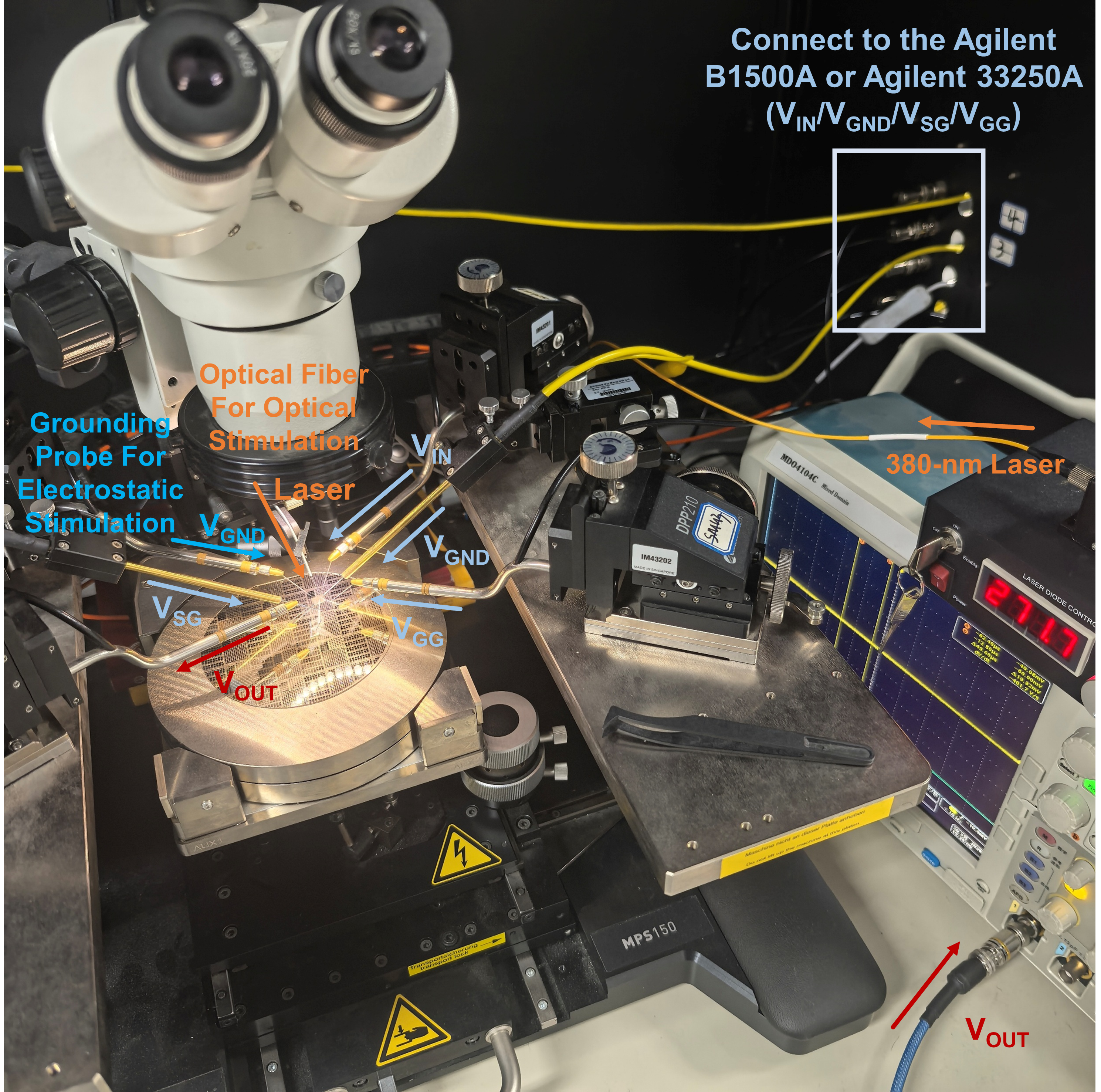


**Figure S17. Test platform for the AMF artificial skin.** The test platform consists of a probe station (including six probes and one optical fiber), a signal generator (Agilent 33250A), a variable resistor, a laser, a semiconductor parameter analyzer (Agilent B1500A), and an oscilloscope. The load voltage on each probe is shown in the photo, where V_IN_ is a square wave signal controlled by the signal generator, and V_SG_/V_GG_/V_GND_ are constant voltage signals controlled by the semiconductor parameter analyzer. Optical stimulation is generated by the laser and applied to a specific region through an optical fiber with a core diameter of 150 μm. The output voltage (V_OUT_) is received by the oscilloscope. The variable resistor is used to adjust the current magnitude of the entire test circuit.


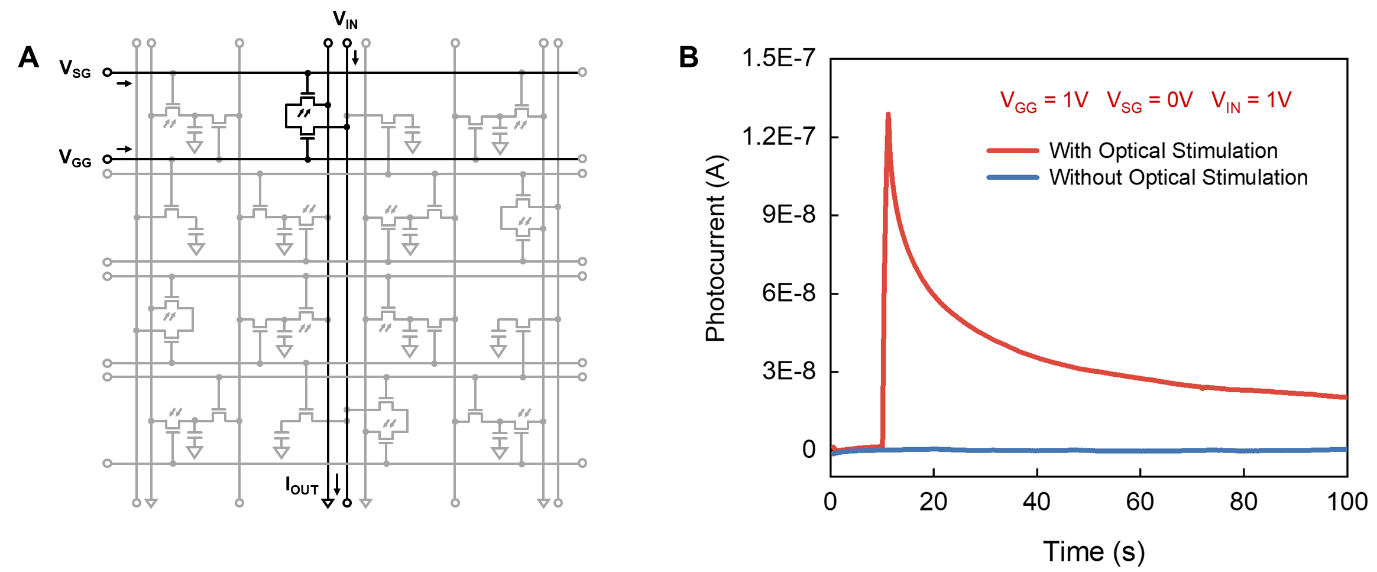


**Figure S18. Measuring circuit and typical response of optical receptors in the AMF artificial skin.** (**A**) Partial equivalent circuit of the AMF artificial skin when an optical receptor is active. The applied V_GG_, V_SG_ and V_IN_ are 1 V, 0 V and 1 V, respectively. (**B**) Photocurrent of the optical receptors in artificial skin with and without optical stimulation (optical power of 50 mW, irradiation time of 1 s). A significant photocurrent is observed upon stimulation.


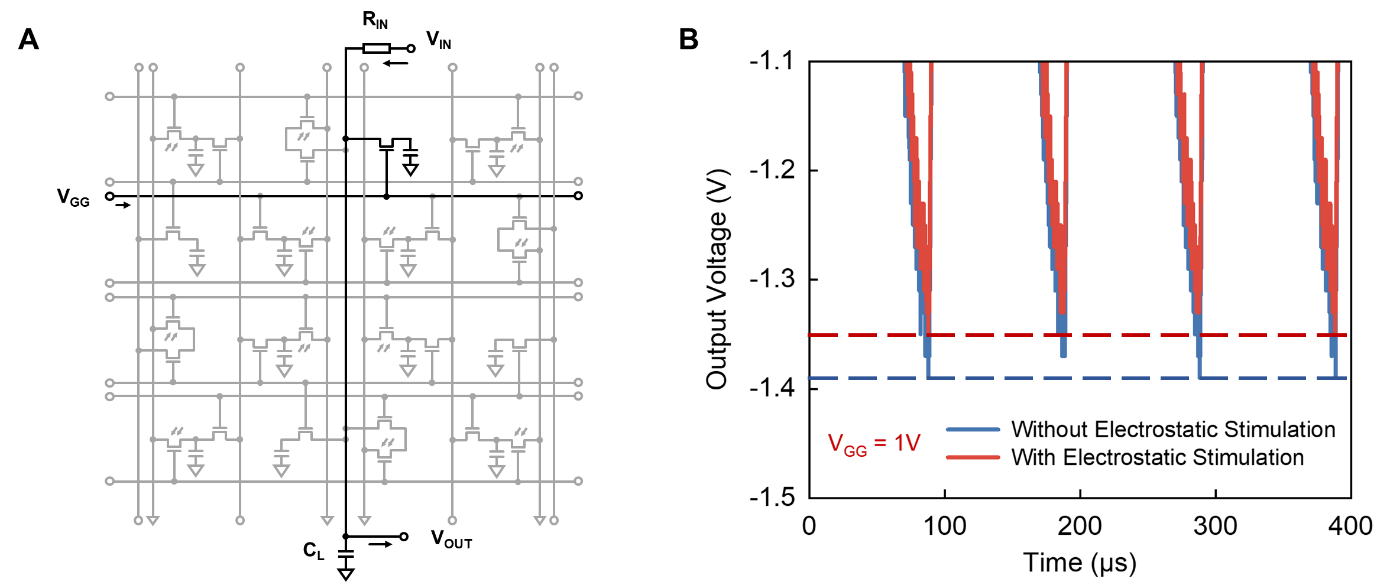


**Figure S19. Measuring circuit and typical response of electrostatic capacitive receptors in the AMF artificial skin.** (**A**) Partial equivalent circuit of the AMF artificial skin when an electrostatic capacitive receptor is active. The V_GG_, R_IN_ and C_L_ are 1 V, 25 kΩ and 1 nF, respectively. The applied input voltage (V_IN_) is a square wave signal with a frequency of 10 kHz, a peak-to-peak voltage of 3 V (average voltage of 0 V) and a duty cycle of 50%. (**B**) Output voltage of the electrostatic capacitive receptors in artificial skin with and without electrostatic stimulation (C_touch_ of 1 nF). A voltage amplitude decrease of approximately 70 mV is observed with electrostatic stimulation.


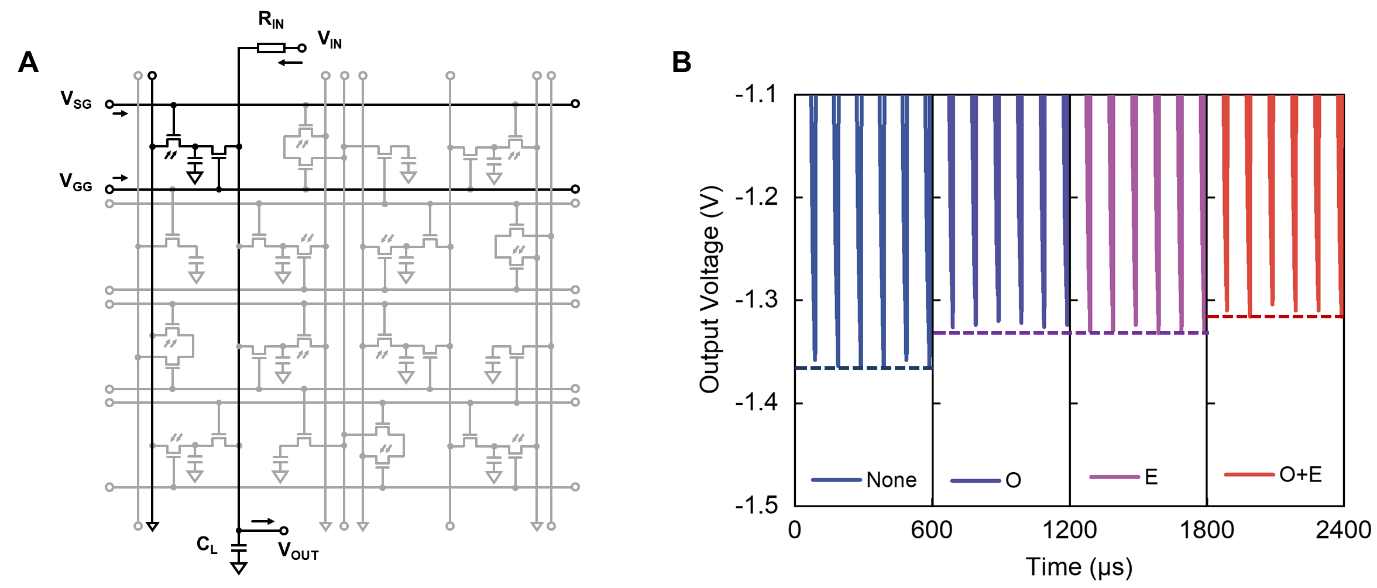


**Figure S20. Measuring circuit and typical response of AMF taxels in the AMF artificial skin.** (**A**) Partial equivalent circuit of the AMF artificial skin when an AMF taxel is active. The V_GG_, V_SG_, R_IN_ and C_L_ are 1 V, 0 V, 25 kΩ and 1 nF, respectively. The applied input voltage (V_IN_) is a square wave signal with a frequency of 10 kHz, a peak-to-peak voltage of 3 V (average voltage of 0 V) and a duty cycle of 50%. (**B**) Output voltage of the AMF taxels in array under varying stimulations. The optical stimulation is 50 mW for 1 s at 380 nm. The electrostatic stimulation introduces C_touch_ of 1 nF. A voltage amplitude decrease of approximately 40 mV is observed with singlemode stimulation, whereas dual-stimulation results in a larger decrease of around 70 mV.


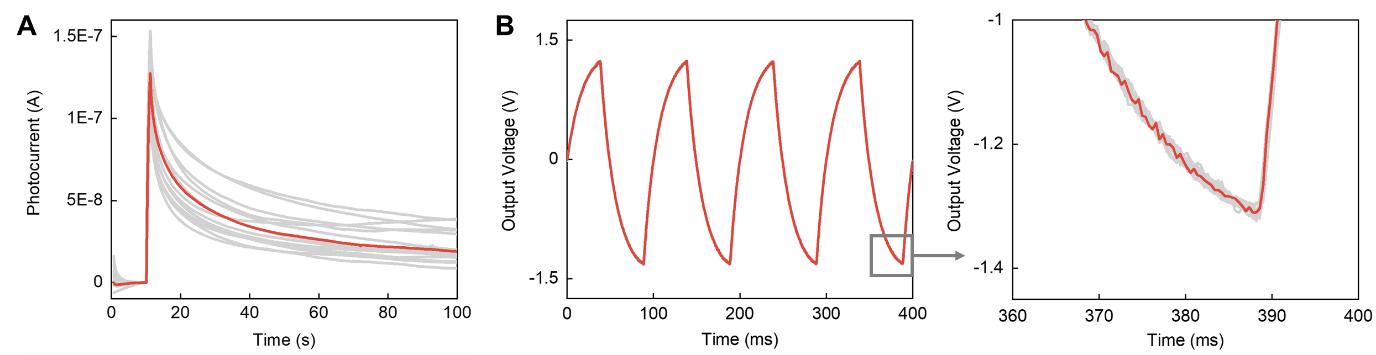


**Figure S21. Device-to-device (D2D) variability of (A) the optical receptors and (B) the electrostatic receptors in the AMF artificial skin.** The grey lines represent the measurements from 14 additional devices across the array. The relative standard deviation (RSD) is calculated as:

$$RSD=\frac{SD}{Mean}*100\% (1)$$

where $SD$ is the standard deviation of the samples and $Mean$ is the sample mean. For the optical response of the optical receptors:

$${Mean}_{o}=\frac{\sum_{i=1}^{n} I_{photo,i}}{n} (2)$$

$${SD}_{o}=\sqrt{\frac{\sum_{i=1}^{n} {(I_{photo,i}-Mean)}^{2}}{n-1}} (3)$$

where $I_{photo,i}$ is the photocurrent peak value of the sample of index i, and $n$ is the number of samples. For the electrostatic response of the electrostatic capacitive receptors:

$${Mean}_{E}=\frac{\sum_{i=1}^{n} {\Delta V}_{i}}{n} (4)$$

$${SD}_{E}=\sqrt{\frac{\sum_{i=1}^{n} {({\Delta V}_{i}-Mean)}^{2}}{n-1}} (5)$$

where ${\Delta V}_{i}$ is the voltage amplitude change of the sample of index i, defined as the difference in amplitude before and after electrostatic stimulation, and $n$ is the number of samples. The final calculated values of the relative standard deviations are: ${RSD}_{O}=14.39\%$ and ${RSD}_{E}=11.37\%$.


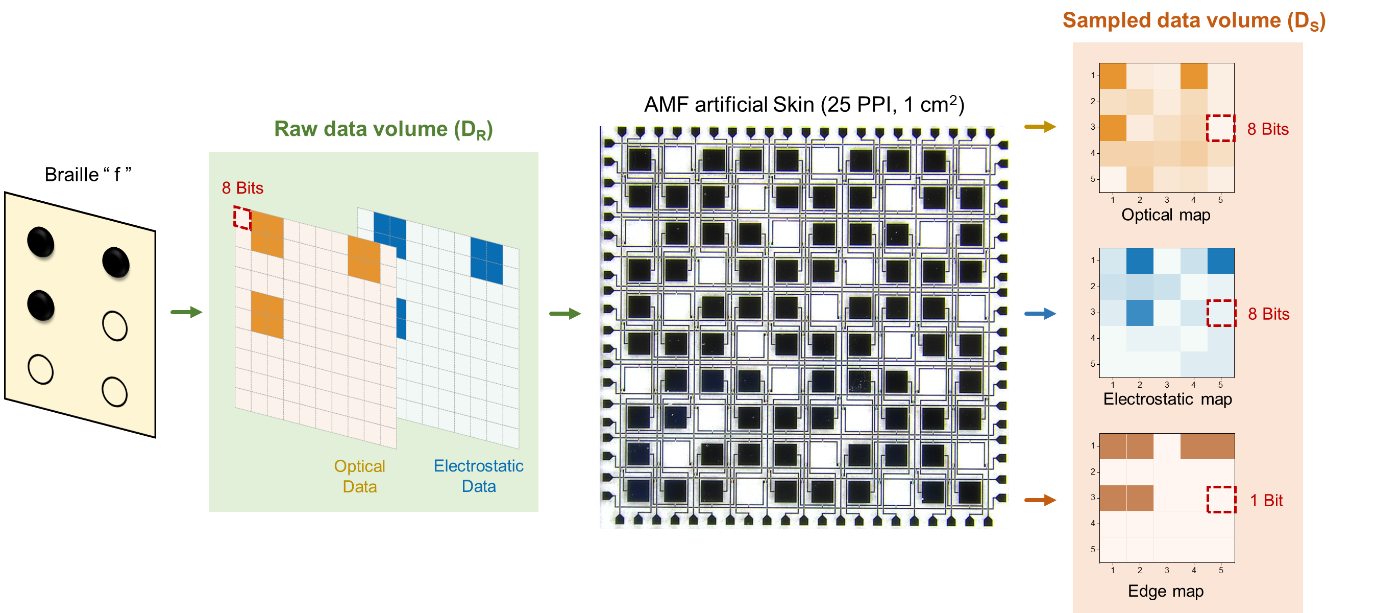


**Figure S22.** **Information compression ratio of the AMF artificial skin.** For a 10×10 AMF artificial skin, the raw data volume can be expressed as:

$$D_{R}=2 \left( channels \right)\times8 \left( bit depth \right)\times10 \left( width \right)\times10 \left( height \right)=1600 Bits (1)$$

The sampled data volume can be expressed as:

$$D_{S}=8 \left( bit depth \right)\times5 \left( width \right)\times5 \left( height \right)+8\times5\times5+1\times5\times5= 425 Bits (2)$$

where the three terms correspond to the optical, electrostatic, and edge maps, respectively. Therefore, the resulting information compression ratio (D_R_/D_S_) is approximately 3.76.


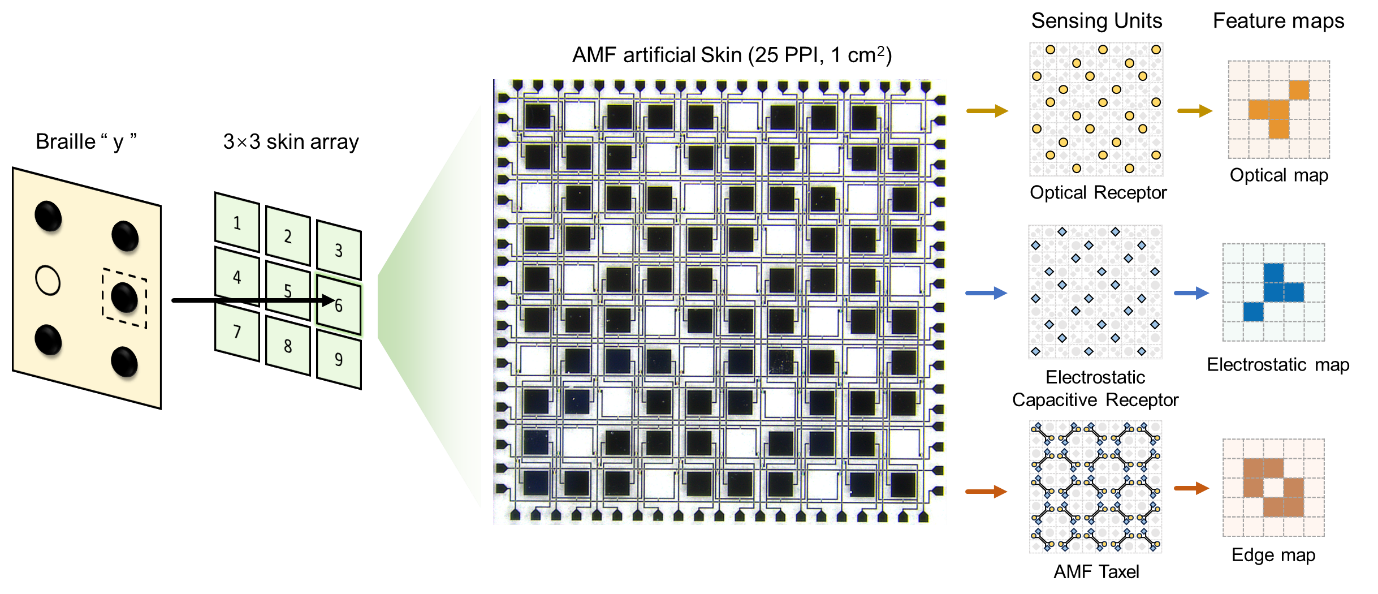


**Figure S23. Schematic flowchart of the perception for enlarged (3×) Braille patterns using a 3 × 3 AMF artificial skin array.** Each Braille dot is enlarged to cover a 4 × 4 pixel area at the center of the skin.

**
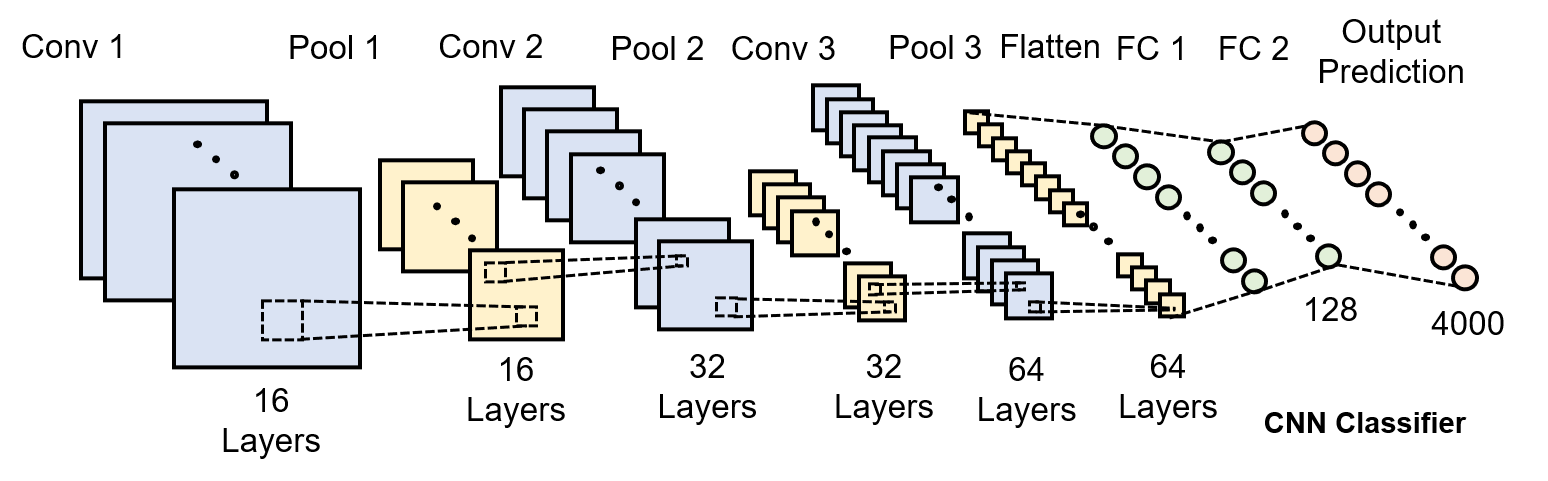
**

**Figure S24. Architecture of the customized CNN classifier for fingerprint-based PIV.** It consists of three convolutional layers (with 16, 32, and 64 output channels), each using 3×3 kernels (stride = 1, padding = 1), followed by a batch normalization layer (BN), an activation function (ReLU), and max-pooling layers (kernel size = 2, stride = 2). Two fully connected layers with 128 and 4000 neurons (dropout rate = 0.2) are used at the end. Batch normalization and ReLU are also applied between the fully connected layers. For the AMF artificial skin, the three feature maps were separately fed into three identical CNN classifiers, and the final classification result was obtained by fusing their outputs via a soft voting strategy, assigning equal weight to each prediction.


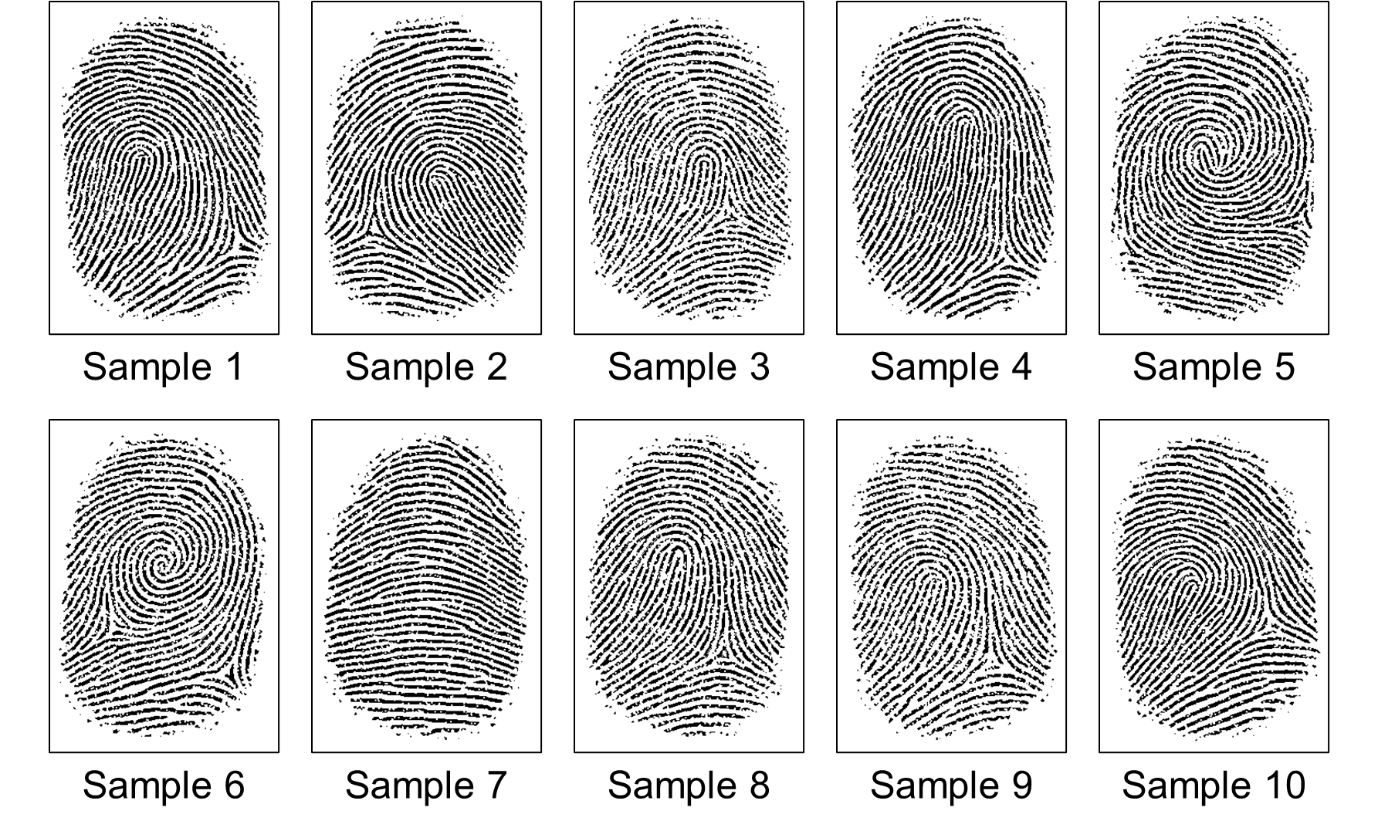


**Figure S25. Ten representative fingerprint samples from the dataset used in the fingerprint-based PIV task.**


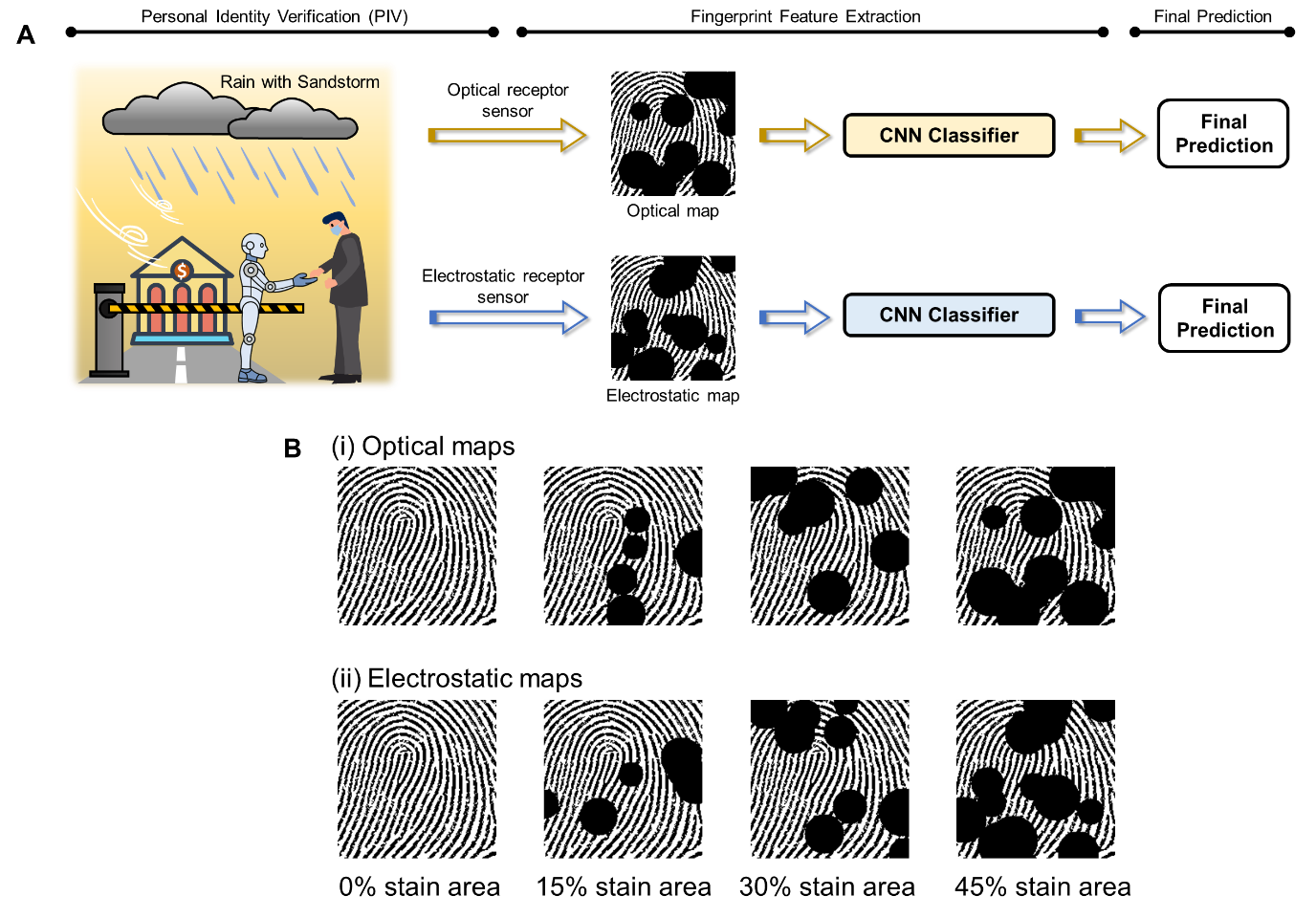


**Figure S26. Singlemode fingerprint recognition under combined rain and sandstorm conditions.** (**A**) Schematic of the fingerprint-based PIV process using conventional singlemode artificial skins (optical or electrostatic) under combined rain and sandstorm conditions. (**B**) Typical fingerprint optical maps (i) and electrostatic maps (ii) extracted by singlemode artificial skins with varying water and mud stain areas (0%, 15%, 30% and 45%) under combined rain and sandstorm conditions. In singlemode artificial skins, each type of receptor is densely arranged, resulting in feature maps that have the same spatial dimensions as the artificial skin itself. Each feature map consists of 192 × 192 pixels.


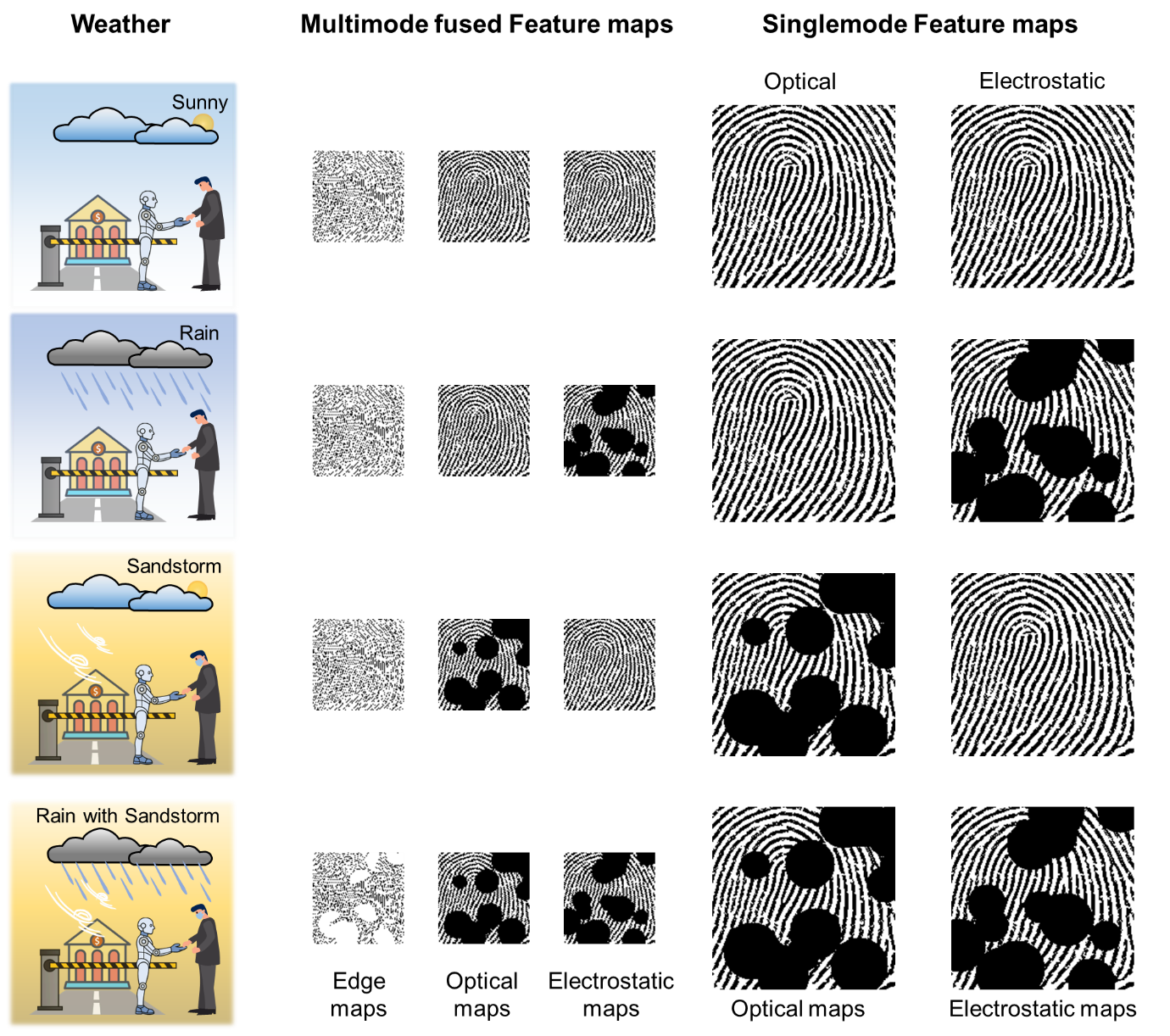


**Figure S27.** **Typical feature maps extracted by the AMF artificial skin and conventional singlemode artificial skins (optical or electrostatic) under different environmental conditions.** Rain primarily degrades the electrostatic maps, while sandstorms mainly impair the optical maps. Notably, edge maps remain unaffected by either type of singlemode interference.


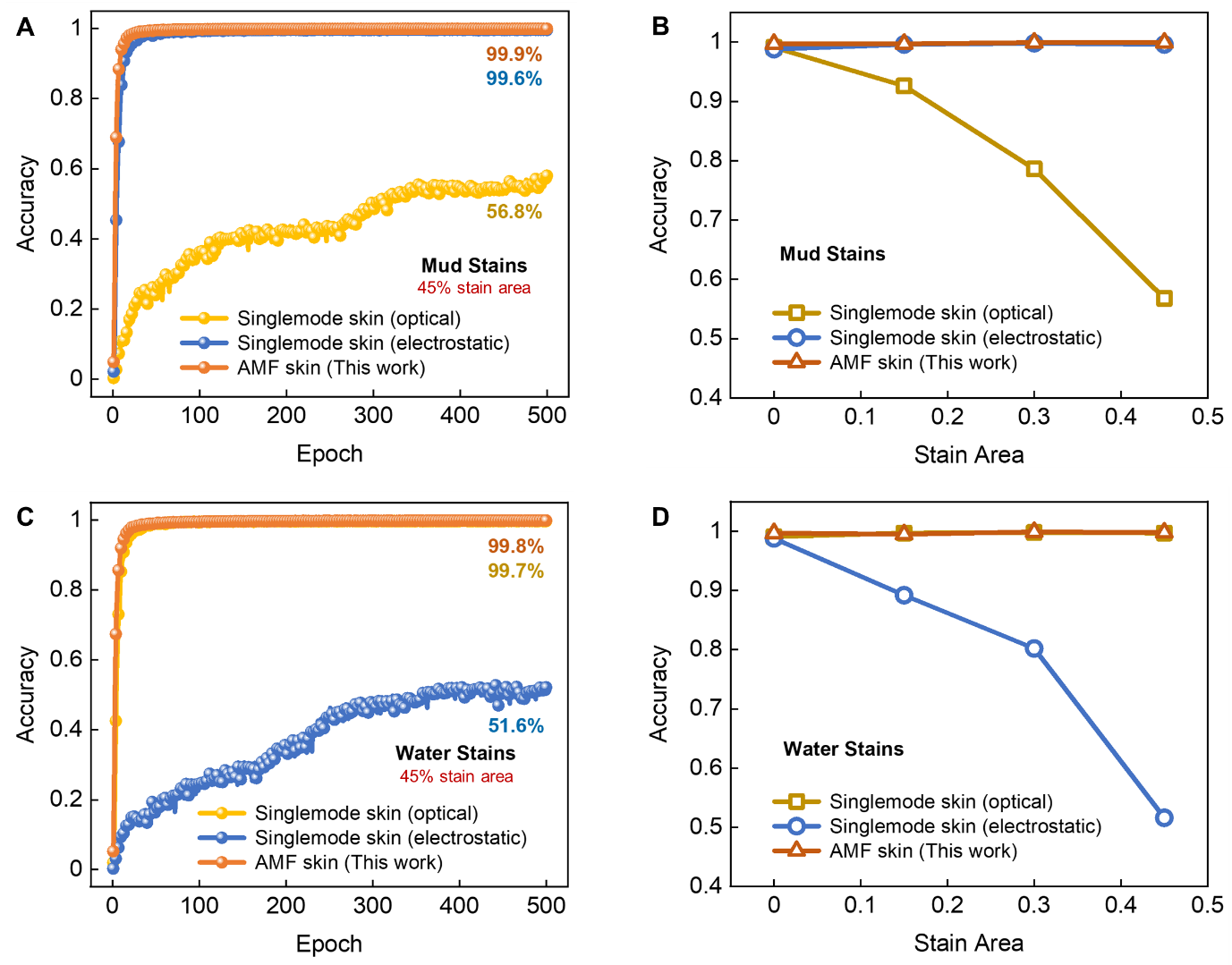


**Figure S28. Summary of fingerprint-based PIV accuracy based on different artificial skins under singlemode interference.** (**A**) PIV accuracy over training epochs based on different artificial skins with 45% mud stain area. (**B**) PIV accuracy of different artificial skins across various mud stain areas (0%, 15%, 30% and 45%). A decrease in accuracy with increasing mud coverage is observed only when using optical artificial skin. (**C**) PIV accuracy over training epochs based on different artificial skins with 45% water stain area. (**D**) PIV accuracy of different artificial skins across various water stain areas (0%, 15%, 30% and 45%). A decrease in accuracy with increasing water coverage is observed only when using electrostatic capacitive artificial skin.


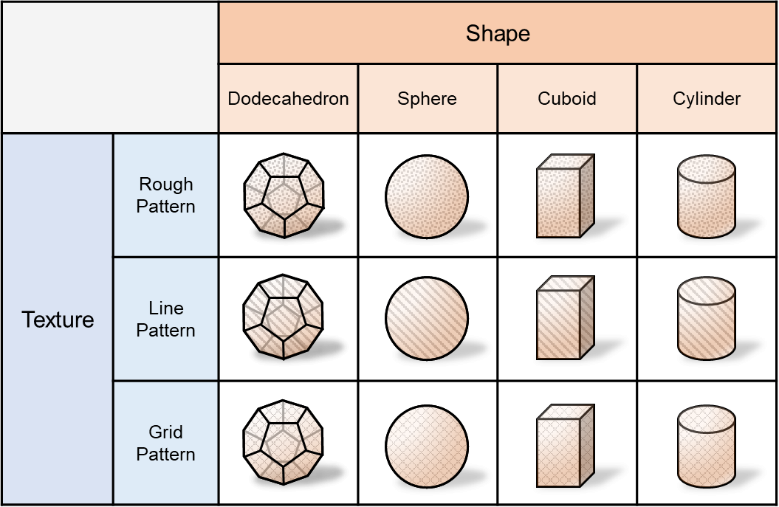


**Figure S29. The twelve distinct object categories used in the object recognition task.**


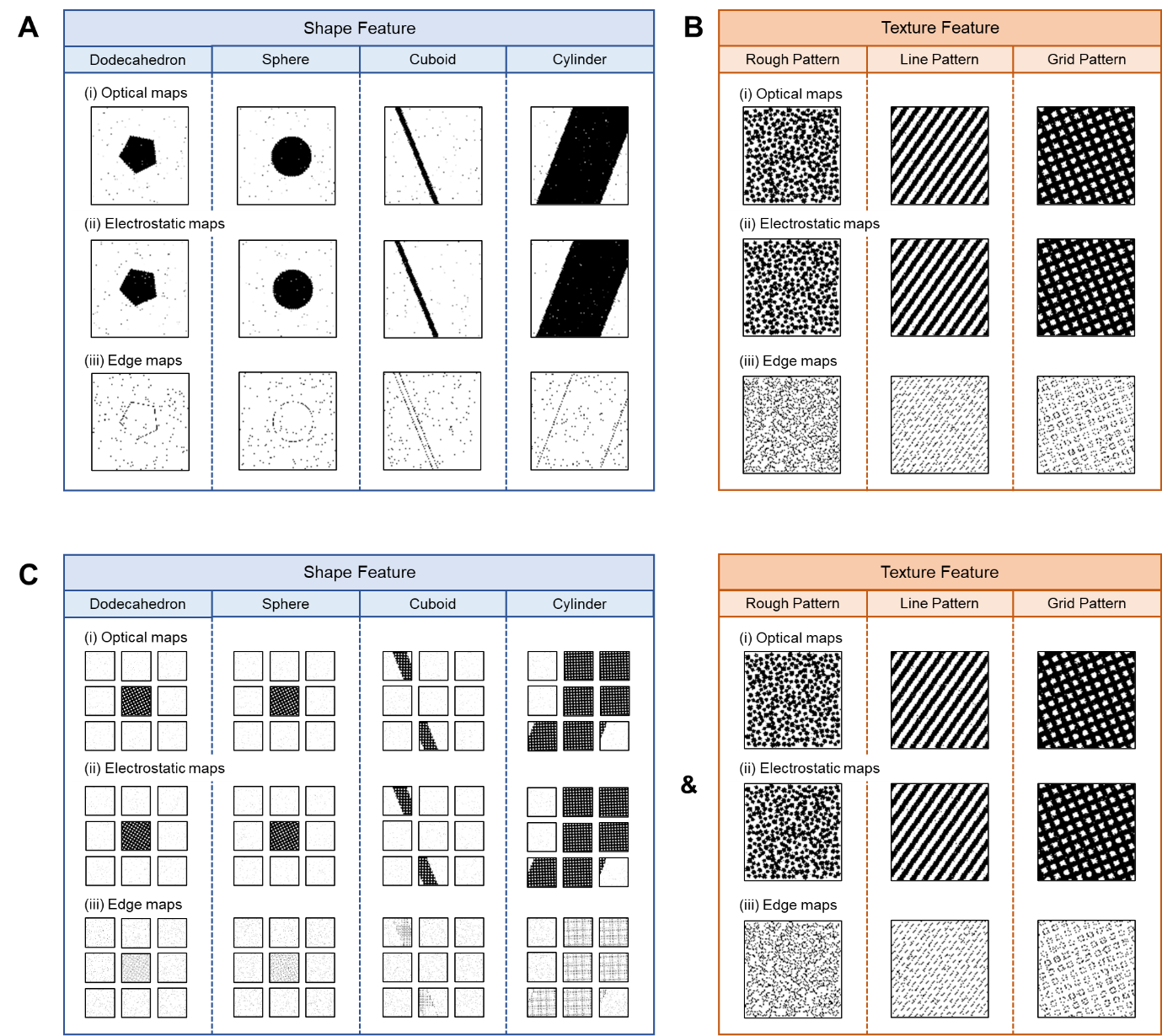


**Figure S30. Typical feature maps extracted by (A) the single low-density tactile system; (B) the single high-density tactile system; and (C) the discrete high-density tactile system.** The single low- or high-density systems produce only 3 shape- or texture-related feature maps, respectively. The discrete high-density system yields 27 shape-related and 3 texture-related feature maps.


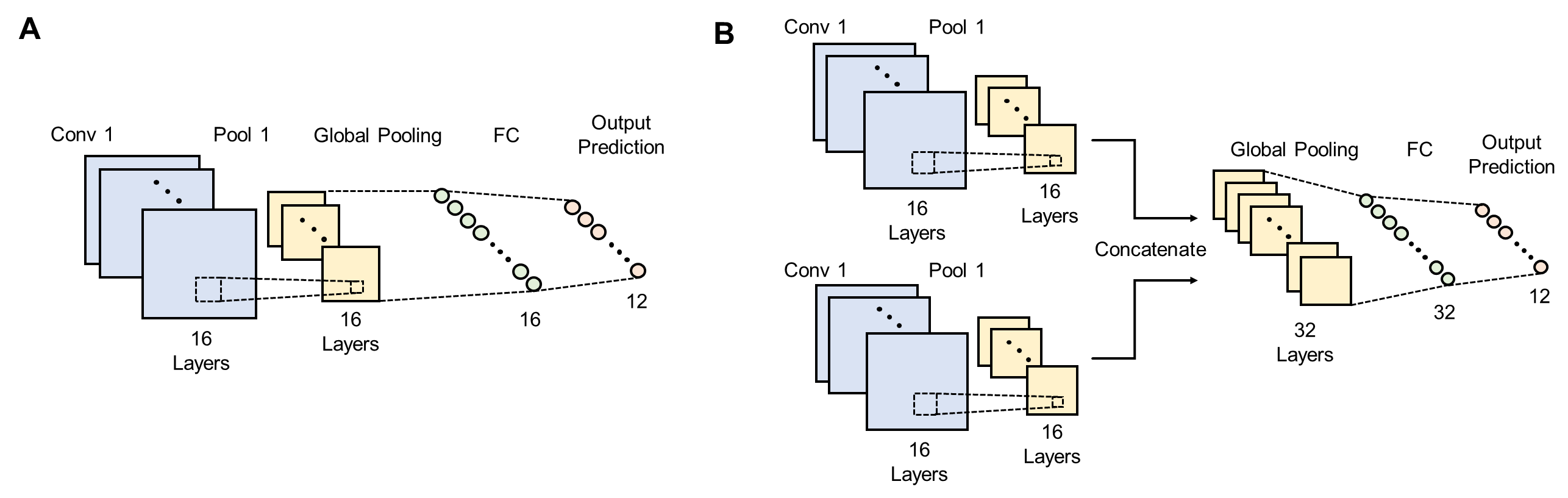


**Figure S31. Architecture of the customized CNN classifiers for object classification.** (**A**) Schematic structure of a single-branch CNN classifier used for single resolution tactile systems. The convolutional layer adopts 3×3 kernels with 16 filters, a stride of 1, and padding of 1. The first pooling layer performs max pooling with a kernel size and stride of 2, while the second pooling layer applies global average pooling. The fully connected layer includes 12 output neurons. A batch normalization (BN) layer followed by a ReLU activation function is inserted between the convolutional and the first pooling layers. (**B**) Schematic structure of a dual-branch CNN classifier for dynamic-taxel-density tactile system and discrete high-density tactile system. The two input branches separately process shape and texture feature maps and are concatenated along the channel dimension after the first pooling layer. All layer parameters are kept identical to those in (A). Three types of feature maps (optical, electrostatic and edge) were separately fed into three identical CNN classifiers, and the final classification result was obtained by fusing their outputs via a soft voting strategy, assigning equal weight to each prediction.


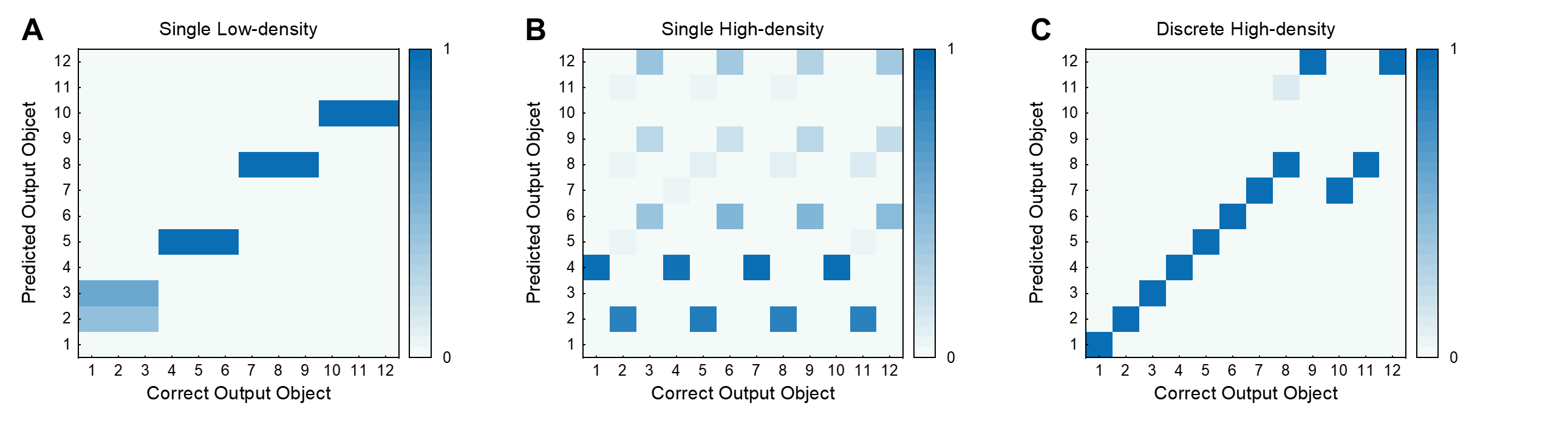


**Figure S32. Confusion matrices of predicted output versus correct output after 100 training epochs for the object recognition task using: (A) the single low-density tactile system; (B) the single high-density tactile system; and (C) the discrete high-density tactile system.**


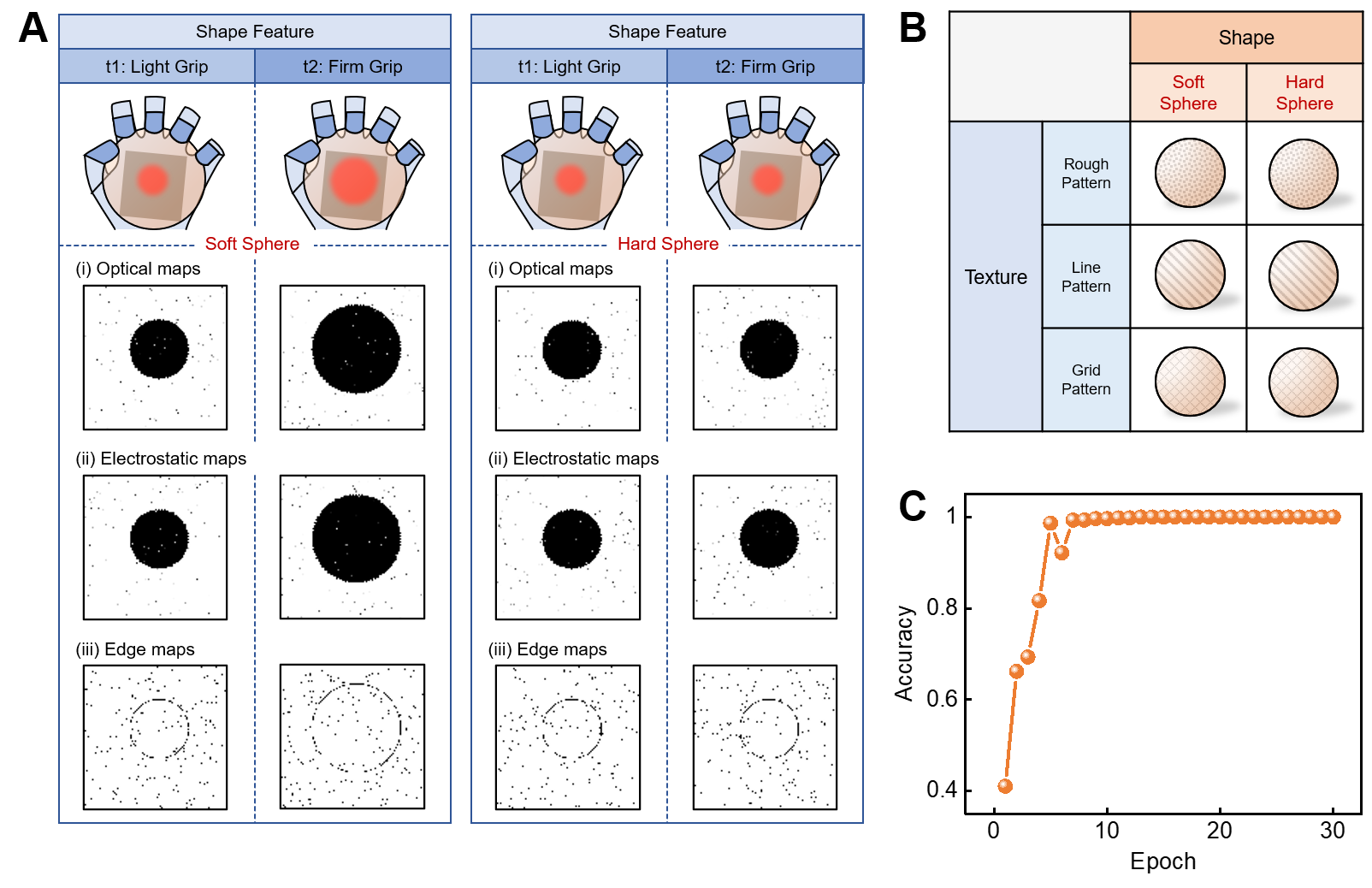


**Figure S33. Dynamic object classification task based on the dynamic-taxel-density tactile system.** (**A**) Typical shape feature maps extracted by the dynamic-taxel-density tactile system during the progressive tightening of the dexterous hand’s grip. Soft objects undergo deformation during this process, resulting in differences in the shape feature maps extracted by the dynamic-taxel-density tactile system at different moments. In contrast, hard objects show negligible differences in the extracted shape feature maps at any time. (**B**) The six distinct object categories used in the dynamic object classification task. (**C**) Classification accuracy across training epochs based on the dynamic-taxel density tactile system.


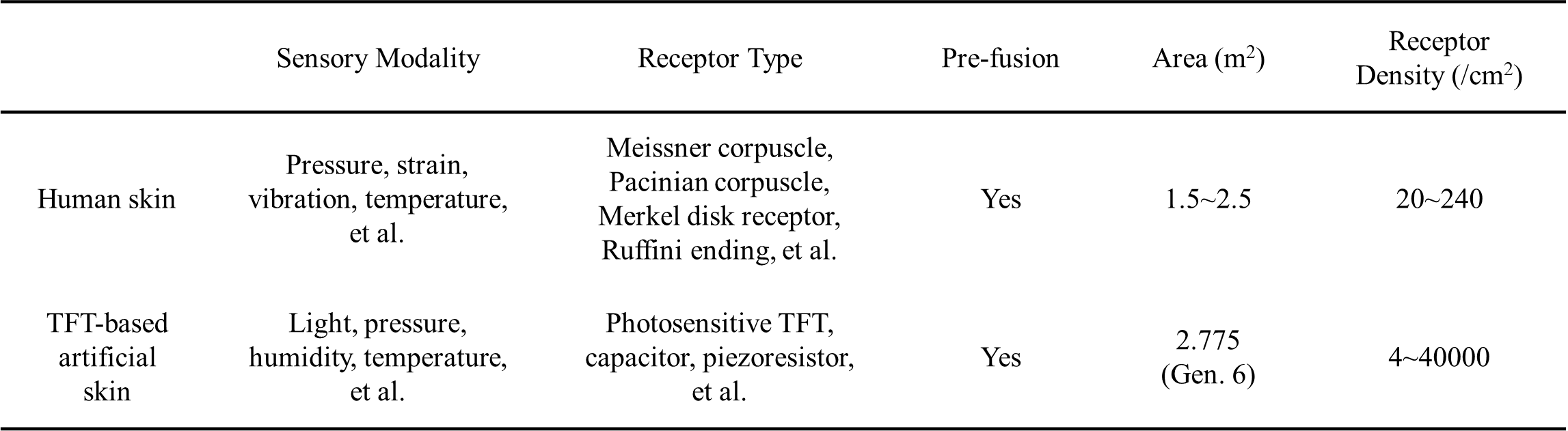


**Table S1. Comparison of the TFT-based artificial skin and human skin.** Human skin contains multiple types of sensory receptors and a large number of free nerve endings, enabling the perception of external physical stimuli such as pressure, stress, vibration, and temperature. Among them, four types of mechanoreceptors—Meissner corpuscles, Pacinian corpuscles, Merkel disk receptors, and Ruffini endings—play a crucial role in tactile perception.^[7]^ These mechanoreceptors convert mechanical stimuli applied to the skin into electrical signals, which first transmit via their respective axons to the spinal cord for preliminary integration and then propagate to the brain to form tactile perception.^[8]^ However, the response characteristics of these receptors to stimuli differ. Notably, individual receptors are typically arranged in an orderly and non-overlapping manner across the skin, while the receptive fields of distinct receptors often overlap.^[9]^ This unique spatial organization enables tactile perception to arise from the superposition and complementation of neural impulses generated by different types of receptors. Despite the fact that the skin is the largest organ of the human body (1.5-2.5 m^2^),^[10]^ the generation and transmission of tactile signals do not incur excessive physiological cost. This efficiency is attributed to the reasonable distribution of receptors and a multimode fused perception strategy. On one hand, receptor density is non-uniform across the skin: high in frequently contacted regions like fingertips (~ 240/cm^2^) and low in areas like the abdomen and back (~ 20/cm^2^), which greatly reduces redundant tactile data.^[11]^ On the other hand, a large portion of tactile information undergoes preliminary fusion in the spinal cord, thereby reducing the processing burden on the brain.


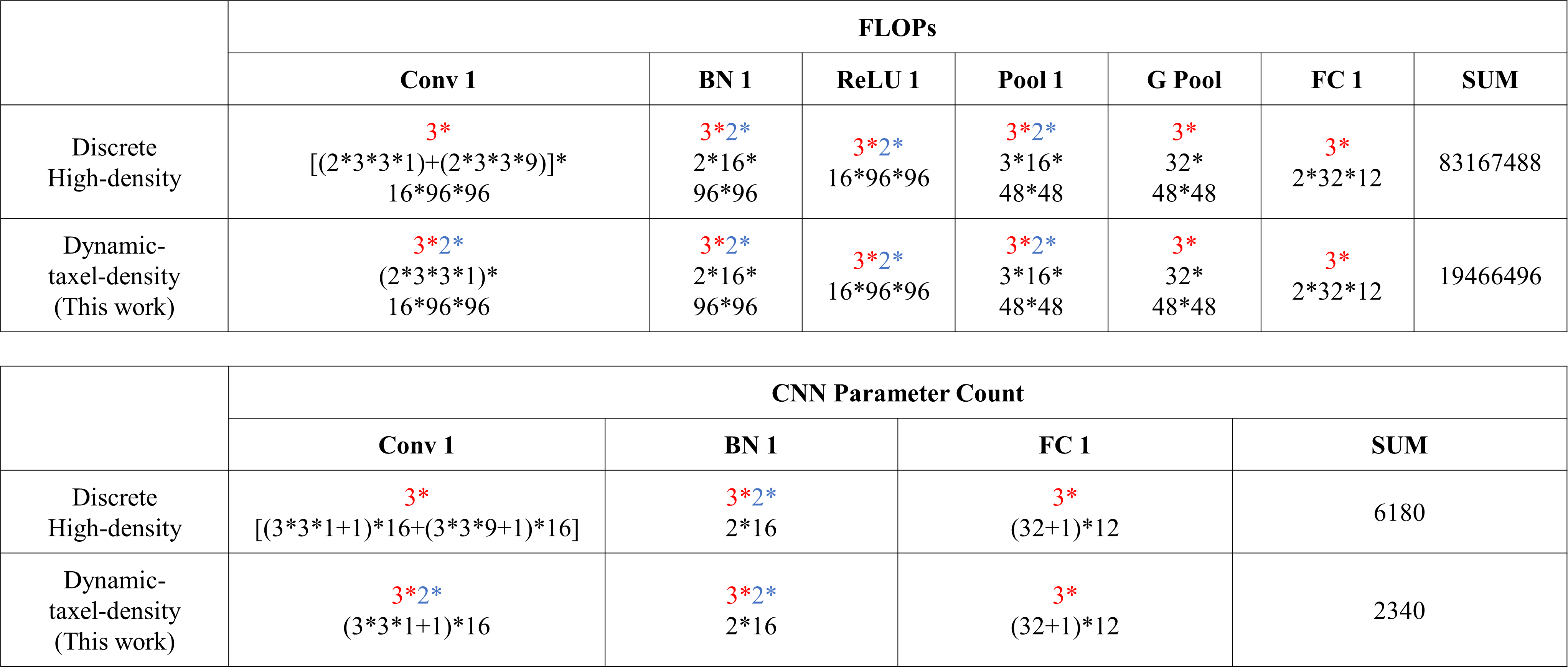


**Table S2. Comparison of data volume, FLOPs, and CNN parameter count between the dynamic-taxel-density and discrete high-density tactile systems.** In terms of data volume, each feature map extracted from both tactile systems has a resolution of 96 × 96 pixels. The data type is uint8, with each pixel occupying 1 byte (8 bits). For the dynamic-taxel-density system, 6 feature maps are generated per recognition task:

$$Data Volume=6*\left( 96*96 \right)*1 byte=54 KB (1)$$

For the discrete high-density system, 30 feature maps are generated:

$$Data Volume=30*\left( 96*96 \right)*1 byte=270 KB (2)$$

In terms of FLOPs, each floating-point addition, multiplication, or comparison in the forward propagation process is considered one FLOP. The FLOPs are calculated based on the components of the CNN classifier.^[12]^ For the convolutional layer:

$$FLOPs=2*F_{W}*F_{H}*C_{IN}*C_{OUT}*W*H (3)$$

For the batch normalization (BN) layer:

$$FLOPs=2*C_{IN} *W*H (4)$$

For the ReLU function:

$$FLOPs=C_{IN} *W*H (5)$$

For the first pooling layer (max pooling):

$$FLOPs=3*C_{IN} *W*H (6)$$

For the global average pooling layer:

$$FLOPs=C_{IN} *W*H (7)$$

For the fully connected layer:

$$FLOPs=2*N_{IN}*N_{OUT} (8)$$

where the $F_{W}$ is the width of the filters, $F_{H}$ is the height of the filters, $C_{IN}$ is the number of input channels, $C_{OUT}$ is the number of output channels, $W$ is the width of the output feature maps, $H$ is the height of the output feature maps, $N_{IN}$ and $N_{OUT}$ are the numbers of neurons in the previous layer and the current layer, respectively. The total FLOPs depend on the size and number of channels in the feature maps. The 3× and 2× scaling factors (highlighted in red and blue, respectively) reflect the use of three CNN classifiers (for optical, electrostatic, and edge features) and two parallel branches. As a result, the CNN for the dynamic-taxel-density system has significantly lower FLOPs (~1.95×10^7^) compared to that of the discrete high-density system (~8.32×10^7^), mainly due to reduced data volume.

In terms of CNN parameter count, since the ReLU activation and the pooling layers have no learnable parameters, the parameter count is determined by the convolutional layer, the BN layer and the fully connected layer. For the convolutional layer:

$$CNN parameter count=(F_{W}*F_{H}*C_{IN}+1)*C_{OUT} (9)$$

For the BN layer (with the scale parameter ($\gamma$) and the shift parameter ($\beta$)):

$$CNN parameter count= 2*C_{IN} (10)$$

For the fully connected layer:

$$CNN parameter count=(N_{IN}+1)*N_{OUT} (11)$$

where the $F_{W}$ is the width of the filters, $F_{H}$ is the height of the filters, $C_{IN}$ is the number of input channels, $C_{OUT}$ is the number of output channels, $N_{IN}$ and $N_{OUT}$ are the numbers of neurons in the previous layer and the current layer, respectively. The 3× and 2× factors account for different types of CNN classifiers and dual-branch structures. Consequently, the CNN for the dynamic-taxel-density system has fewer parameters (2244) than that for the discrete high-density system (6084), due to the lower input channels.


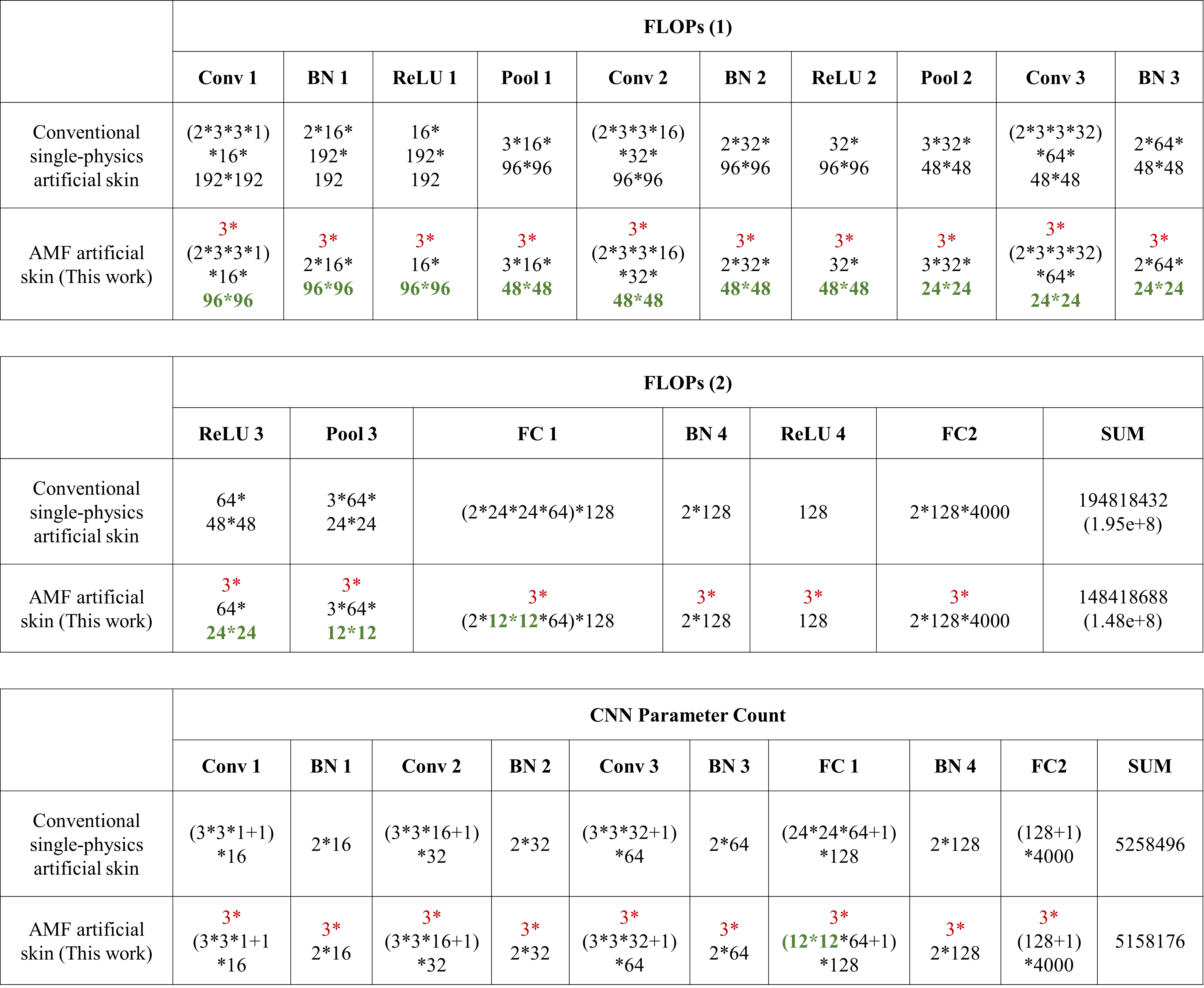


**Table S3. Comparison of data volume, FLOPs, and CNN parameter count between the AMF artificial skin and the conventional singlemode artificial skin.** In terms of data volume, the feature maps are of type uint8, with each pixel occupying 1 byte (8 bits). For the AMF artificial skin, three feature maps (96 × 96 pixels each) are generated per recognition task:

$$Data Volume=3*\left( 96*96 \right)*1 byte=27 KB (1)$$

In contrast, the conventional singlemode artificial skin generates a single feature map of size 192 × 192 pixels:

$$Data Volume=1*\left( 192*192 \right)*1 byte=36 KB (2)$$

Therefore, the AMF artificial skin achieves a 25% reduction in data volume.

In terms of FLOPs, the fundamental expressions follow those defined in Table S2. The 3× factor (highlighted in red) accounts for the use of three CNN classifiers (for optical, electrostatic, and edge features). In summary, the AMF artificial skin achieves an approximately 23.8% reduction in FLOPs compared to the conventional singlemode artificial skin, due to its smaller feature map size.

In terms of CNN parameter count, the basic expressions also follow Table S2. The 3× factor (highlighted in red) accounts for the use of different CNN classifiers. Although the parameter count in convolutional layers is independent of feature map size, over 99% of the total parameters reside in the fully connected layers, where the number of input neurons directly depends on the size and number of channels in the final feature maps. Therefore, the AMF artificial skin achieves an approximately 1.9% reduction in CNN parameter count compared to the conventional system, owing to its smaller feature map size.

**References**

1 Crandall, R. S. Defect relaxation in amorphous silicon: Stretched exponentials, the Meyer-Neldel rule, and the Staebler-Wronski effect. *Physical Review B* **43**, 4057-4070 (1991). https://doi.org:10.1103/PhysRevB.43.4057

2 Chen, Y. F. & Huang, S. F. Connection between the Meyer-Neldel rule and stretched-exponential relaxation. *Physical Review B* **44**, 13775-13778 (1991). https://doi.org:10.1103/PhysRevB.44.13775

3 Li, S., Wang, M., Zhang, D., Wang, H. & Shan, Q. A Unified Degradation Model of a-InGaZnO TFTs Under Negative Gate Bias With or Without an Illumination. *IEEE Journal of the Electron Devices Society* **7**, 1063-1071 (2019). https://doi.org:10.1109/JEDS.2019.2946383

4 Hossain Chowdhury, M. D., Migliorato, P. & Jang, J. Temperature dependence of negative bias under illumination stress and recovery in amorphous indium gallium zinc oxide thin film transistors. *Applied Physics Letters* **102**, 143506 (2013). https://doi.org:10.1063/1.4801762

5 Luo, J. *et al.* Transient photoresponse in amorphous In-Ga-Zn-O thin films under stretched exponential analysis. *Journal of Applied Physics* **113**, 153709 (2013). https://doi.org:10.1063/1.4795845

6 Gupta, D., Yoo, S., Lee, C. & Hong, Y. Electrical-Stress-Induced Threshold Voltage Instability in Solution-Processed ZnO Thin-Film Transistors: An Experimental and Simulation Study. *IEEE Transactions on Electron Devices* **58**, 1995-2002 (2011). https://doi.org:10.1109/TED.2011.2138143

7 Deflorio, D., Di Luca, M. & Wing, A. M. Skin and Mechanoreceptor Contribution to Tactile Input for Perception: A Review of Simulation Models. *Frontiers in Human Neuroscience* **16**, 862344 (2022). https://doi.org:10.3389/fnhum.2022.862344

8 Abraira, Victoria E. & Ginty, David D. The Sensory Neurons of Touch. *Neuron* **79**, 618-639 (2013). https://doi.org:https://doi.org/10.1016/j.neuron.2013.07.051

9 Handler, A. & Ginty, D. D. The mechanosensory neurons of touch and their mechanisms of activation. *Nature Reviews Neuroscience* **22**, 521-537 (2021). https://doi.org:10.1038/s41583-021-00489-x

10 Verbraecken, J., Van de Heyning, P., De Backer, W. & Van Gaal, L. Body surface area in normal-weight, overweight, and obese adults. A comparison study. *Metabolism* **55**, 515-524 (2006). https://doi.org:https://doi.org/10.1016/j.metabol.2005.11.004

11 Corniani, G. & Saal, H. P. Tactile innervation densities across the whole body. *Journal of Neurophysiology* **124**, 1229-1240 (2020). https://doi.org:10.1152/jn.00313.2020

12 Asperti, A., Evangelista, D. & Marzolla, M. in *Machine Learning, Optimization, and Data Science.* (eds Giuseppe Nicosia *et al.*) 86-100 (Springer International Publishing).
